# Design and Synthesis of Dy_2_TmSbO_7_/BiHoO_3_ Heterojunction: The Mechanism and Application for Photocatalytic Degradation of Sulphamethoxypyridazine

**DOI:** 10.3390/molecules31010024

**Published:** 2025-12-22

**Authors:** Jingfei Luan, Minghe Ma, Liang Hao, Hengchang Zeng, Anan Liu

**Affiliations:** 1 School of Physics, Changchun Normal University, Changchun 130032, China; 13251704137@139.com (M.M.); 19845486007@139.com (L.H.); zenghc23@mails.jlu.edu.cn (H.Z.); ananliu2001@outlook.com (A.L.); 2 State Key Laboratory of Pollution Control and Resource Reuse, School of the Environment, Nanjing University, Nanjing 210093, China

**Keywords:** Dy_2_TmSbO_7_/BiHoO_3_ heterojunction, sulphamethoxypyridazine, sulfonamide, visible light absorption, photocatalytic activity

## Abstract

A novel Z-scheme Dy_2_TmSbO_7_/BiHoO_3_ heterostructure photocatalyst was synthesized with the ultrasound-assisted solvothermal method. The Dy_2_TmSbO_7_/BiHoO_3_ heterojunction photocatalyst (DBHP) reflected wonderful separation efficiency of photogenerated electrons and photogenerated holes owing to the efficient direct Z-scheme heterojunction structure characteristic. The lattice parameter and the bandgap energy of the Dy_2_TmSbO_7_ were 10.52419 Å and 2.58 eV, simultaneously, the lattice parameter and the bandgap energy of the BiHoO_3_ were 5.42365 Å and 2.25 eV, additionally, the bandgap energy of the DBHP was 2.32 eV. Above results indicated that DBHP, Dy_2_TmSbO_7_ or BiHoO_3_ possessed an excellent ability for absorbing visible light energy, therefore, DBHP, Dy_2_TmSbO_7_ or BiHoO_3_ owned superior photocatalytic activity for degrading the sulphamethoxypyridazine (SMP) under visible light irradiation. The removal rate of the SMP after visible light irradiation of 135 min with the DBHP was 99.47% for degrading the SMP during the photocatalytic degradation (PADA) process, correspondingly, the removal rate of the total organic carbon (TOC) concentration after visible light irradiation of 135 min with the DBHP was 98.02% for degrading the SMP during the PADA process. The removal rate of the SMP after visible light irradiation of 135 min with the DBHP was 1.15 times, 1.29 times or 2.60 times that with Dy_2_TmSbO_7_, BiHoO_3_ or nitrogen-doped TiO_2_ (N-T). Therefore, the DBHP displayed higher photocatalytic activity for degrading the SMP under visible light irradiation compared with Dy_2_TmSbO_7_, BiHoO_3_ or N-T. Specifically, the mineralization rate for removing the TOC concentration during the PADA process of the SMP with the DBHP was 1.18 times, 1.32 times or 2.79 times that with Dy_2_TmSbO_7_, BiHoO_3_ or N-T. In addition, the stability and reusability of the DBHP were systematically evaluated, confirming that the DBHP owned potential applicability for degrading the antibiotic pollutant, which derived from the practical industrial wastewater. Trapping radicals experiments and the electron paramagnetic resonance measurement experiments were conducted for identifying the reactive radicals, such as the hydroxyl radicals (•OH), the superoxide anions (•O_2_^−^) and the photogenerated holes (h^+^), which were generated with the DBHP for degrading the SMP during the PADA process under visible light irradiation, as a result, the •O_2_^−^ possessed the maximal oxidative capability compared with the •OH or the h^+^. Above results indicated the degradation mechanism and the degradation pathways which were related to the SMP. In conclusion, this study makes a significant contribution for the development of the efficient Z-scheme heterostructure photocatalysts and provides a key opinion to the development of the sustainable remediation method with the view of mitigating the antibiotic pollution.

## 1. Introduction

With the development of the pharmaceutical industry, antibiotics are widely used. Due to the lack of appropriate usage guidelines, the consumption of antibiotics is high in Asia and Africa. In the United States, sulfonamides account for 2.3% of the total wasted antibiotics, and in the European Union, sulfonamides represent the second most used veterinary antibiotics. In Kenya, sulfonamides constitute 22% of the active antimicrobial agents utilized for the production of animal food [1,2]. Specifically, according to the Infectious Diseases Society of America, “trimethoprim–sulfamethoxazole” remains a primary therapeutic option for uncomplicated urinary tract infections [3]. Sulfamethoxazole is widely recognized for its application in treating bacterial infections such as urinary tract infections and prostatitis [4]. Sulfadiazine is extensively utilized in animal growth promotion [5]. Concurrently, antibiotics can effectively prevent and treat bacterial infectious diseases in animals [6,7,8]. Sulphamethoxypyridazine (SMP) is a type of sulfonamides; meanwhile, SMP has been utilized in the clinical treatment of humans and animals in recent years [9,10]. Nevertheless, SMP is incompletely metabolized in organisms, and 30–90% of SMP enters water bodies through excreta; as a result, SMP and the metabolite cause water pollution. SMP is an amphoteric polar substance and is readily soluble in water. Consequently, SMP exhibits high mobility in a water environment. Meanwhile, SMP is constantly detected in surface waters [11]. In addition, the long-term presence of SMP which is contained in water environments could also impact the health of organisms and even induce genetic mutations [12]. Therefore, it is imperative to remove SMP from water environments effectively and to purify water resources.

Studies have found that existing methods have a certain effect on degrading SMP in wastewater. However, existing methods, such as biological methods, physical methods, and chemical methods, all have certain limitations. The principle for purifying wastewater containing SMP that is treated through the biological method involves introducing microorganisms into the pharmaceutical wastewater and providing the corresponding microorganisms with suitable conditions. In order to sustain the survival of the microorganisms, these microorganisms break down the organic pollutants that are present in wastewater. However, maintaining the optimal conditions for microbial activity remains a significant challenge [13]. The physical method for treating SMP involves the application of physical techniques for separating hazardous substances and residues that stem from the pharmaceutical effluent. Since SMP contaminant is solely separated and not eliminated, the collected hazardous concentrates should undergo further processing for ultimate removal [14]. The chemical method for treating SMP utilizes redox reactions to convert SMP pollutants that derive from the pharmaceutical wastewater into other substances; nevertheless, there are risks of high cost and the introduction of additional toxic pollutants into the water [15]. In addition, the industrial progress has resulted in increasing daedal wastewater components, posing challenges to the impactful degradation of the organic pollutants. Therefore, more effective water treatment technologies must be developed and combined with traditional methods for degrading SMP contained in polluted water resources [16].

Studies show that semiconductor photocatalytic technology can remove pollutants from wastewater. As a semiconductor photocatalyst, TiO_2_ can generate photogenerated electrons and photogenerated holes under solar light radiation [17,18,19,20,21]. These photogenerated electrons and photogenerated holes can activate H_2_O and O_2_ for generating •OH and •O_2_^−^; therefore, pollutants that originate from wastewater are degraded effectively [22,23,24,25,26]. The metal-oxide semiconductor photocatalysts possess extremely low utilization efficiency of the solar spectrum energy; nevertheless, only the ultraviolet light energy of the solar spectrum energy can be used. It was found that the A_2_B_2_O_7_-type photocatalyst could utilize the visible light energy of the solar spectrum energy [27,28,29]. For example, Huang et al. reported that the photocatalytic removal rate of the rhodamine B with the Bi_2_Sn_2_O_7_ powder was 87% under visible light irradiation [30]. Similarly, Xu et al. demonstrated that the photocatalytic removal efficiency of the rhodamine B with the La_2_Zr_2_O_7_ nanophase catalyst was 73% under visible light irradiation [31]. However, the existing A_2_B_2_O_7_-type photocatalyst possesses a relatively low utilization efficiency of the solar spectrum energy. Therefore, research efforts have focused on structural modification of the A_2_B_2_O_7_-type photocatalyst. The Gd_2_Zr_2_O_7_ pyrochlore structure photocatalyst is an example of an A_2_B_2_O_7_-type photocatalyst [32]. In research on TiO_2_, San et al. made a further contribution by synthesizing Dy-doped TiO_2_, which exhibited a similar enhancement for the photocatalytic degradation (PADA) of methylene blue compared with the undoped TiO_2_ [33]. In accordance with the investigation’s results, which were gained by Mazierski et al., the Tm-doped TiO_2_, which was prepared by Mazierski et al., showed significantly enhanced photodegradation efficiency of the phenol compared with the pristine TiO_2_ [34]. Jooho et al. also synthesized Sb-doped TiO_2_, which showed enhanced degradation efficiency of the phenol compared with the undoped TiO_2_ [35]. Heshan et al. synthesized the Ho-doped TiO_2_; concurrently, Heshan et al. observed that the Ho-doped TiO_2_ possessed a significant increase in the photodegradation efficiency of the methyl orange compared with the pure TiO_2_ [36]. In addition, Xu et al. presented research results which were obtained using synthetic Bi-doped TiO_2_, revealing that the Bi-doped TiO_2_ owned significant improvements in the degradation efficiency of the rhodamine B and the phenol compared with the pure TiO_2_ [37]. In general, the above studies emphasized the efficacy of the incorporated elements such as Dy, Tm, and Sb for enhancing the photocatalytic activity of the TiO_2_; meanwhile, the incorporating efficacy of the Bi element and the Ho element was to improve the photocatalytic activity of the TiO_2_. Based on abovementioned findings, we proposed to use Dy for replacing Gd, which stemmed from Gd_2_Zr_2_O_7_; simultaneously, Tm could replace one Zr, which derived from Gd_2_Zr_2_O_7_; ultimately, Sb might replace another Zr, which originated from Gd_2_Zr_2_O_7_; therefore, a new single-phase photocatalyst Dy_2_TmSbO_7_ was prepared. Studies have demonstrated that the fluorite structure photocatalyst BiGdO_3_ can effectively utilize visible light energy, which derives from the solar spectrum energy [38]. In order to intensify the photocatalytic activity, we modified the structure of the BiGdO_3_ photocatalyst by substituting Gd, which stemmed from the BiGdO_3_ photocatalyst with Ho; thereby, a novel single-phase photocatalyst BiHoO_3_ could be fabricated.

However, the challenge of a high recombination rate for the photogenerated electrons and the photogenerated holes, which stem from the single-component photocatalyst and light irradiation, remain prominent. Consequently, the binary heterostructure photocatalysts are gaining increasing popularity; simultaneously, the interfaces that exist between the two components generate internal electric fields, which facilitate the separation of the photogenerated electrons and the photogenerated holes. Currently, the Z-scheme heterojunction photocatalysts possess superior photocatalytic activity compared with the traditional type-II heterojunction photocatalysts; accordingly, the Z-scheme heterojunction photocatalysts have attracted greater attention.

In the Z-scheme heterojunction photocatalyst system, the photogenerated electrons are retained in the more negative conduction band of the Z-scheme heterojunction photocatalyst; meanwhile, the photogenerated holes are retained in the more positive valence band of the Z-scheme heterojunction photocatalyst. Nevertheless, the ineffective photogenerated electrons and the photogenerated holes are recombined. As a result, a greater redox potential that is higher than the redox potential of the type-II heterojunction photocatalyst was achieved [39,40,41,42]. Therefore, the photocatalytic activity of the Z-scheme heterojunction photocatalyst could be improved by accelerating the migration rate of the photogenerated electrons and the photogenerated holes; meanwhile, the combination of the photogenerated electrons and the photogenerated holes and aquatic dissolved oxygen and hydroxyl would exhibit strong redox ability. It was worth noting that the Z-scheme heterojunction photocatalyst have made important progress in the field of the photocatalysts. For instance, Juanqin et al. structured a direct Z-scheme Bi_2_MoO_6_/BiOCl hybrid for increasing the PADA efficiency of the ciprofloxacin under visible light irradiation [43]. Huang et al. also prepared a direct Z-scheme Ag_2_CO_3_/ZnFe_2_O_4_ heterojunction photocatalyst, which exhibited excellent photocatalytic activity for degrading norfloxacin [44]. The above analysis results indicate that a Z-scheme heterojunction photocatalyst was introduced for promoting the charge separation; as a result, the photocatalytic activity of the Z-scheme heterojunction photocatalyst could be enhanced significantly. In accordance with the aforementioned analysis results, a Z-scheme heterojunction photocatalyst was constructed for facilitating the separation of the photogenerated electrons and the photogenerated holes and had higher photocatalytic activity [45,46,47,48,49,50].

We were inspired by the above constructive opinions and guided by suitable energy band structures; thus, a novel direct Z-scheme Dy_2_TmSbO_7_/BiHoO_3_ heterojunction photocatalyst (DBHP) was developed for the efficient removal of SMP that was contained in pharmaceutical wastewater under the condition of visible light irradiation. This photocatalyst design leveraged the Z-scheme heterojunction structure and facilitated the separation of photogenerated electrons and photogenerated holes; thereby, the photocatalytic activity of the DBHP was enhanced for degrading SMP. The combination of Dy_2_TmSbO_7_ component and the BiHoO_3_ component that remained with the heterojunction photocatalyst Dy_2_TmSbO_7_/BiHoO_3_ could enable visible light energy to be utilized effectively during the PADA process of SMP. The experimental validation confirmed that DBHP possessed higher photocatalytic activity; as a result, a removal rate of 99.47% for degrading SMP was achieved using DBHP after visible light irradiation of 135 min. This significant advancement underscored the superior efficacy of DBHP compared with other traditional photocatalysts which were employed for degrading SMP under visible light irradiation.

In this study, we utilize a series of comprehensive characterization techniques, including X-ray diffraction pattern, ultraviolet and visible absorption spectrum, Fourier transform infrared spectrum, Raman spectrogram, X-ray photoelectron spectrum, transmission electron microscopy image, energy-dispersive X-ray spectrum, photoluminescence spectrogram, photocurrent intensity measurement spectrum, electrochemical impedance spectrogram, ultraviolet photoelectron spectrogram, and electron paramagnetic resonance spectrum. Concurrently, the above spectra were utilized for analyzing the morphological traits, composition content, photochemistry characteristics, and photoelectric properties of the pure phase of the Dy_2_TmSbO_7_ photocatalyst, pure phase of the BiHoO_3_ photocatalyst, and the DBHP. In addition, the PADA efficiency of SMP that stems from pharmaceutical wastewater using DBHP, Dy_2_TmSbO_7_, BiHoO_3_, or nitrogen-doped TiO_2_ (N-T) was systematically evaluated under visible light irradiation. A vital contribution of this study is the pathbreaking synthesis technique for preparing a visible-light-responsive Dy_2_TmSbO_7_ photocatalyst and a visible-light-responsive BiHoO_3_ photocatalyst using a solid-state calcination method. In addition, this study successfully prepared a Z-scheme heterojunction photocatalyst in accordance with elemental composition variation in the A_2_B_2_O_7_ photocatalyst for the first time using an ultrasound-assisted solvothermal method. Meanwhile, a series of photodegradation experiments using the newly synthesized photocatalysts under visible light irradiation in this study were conducted for evaluating and comparing the photocatalytic activity of the photocatalysts which were reported in our previous studies. Notably, the Dy_2_TmSbO_7_, BiHoO_3_, and DBHP exhibited significantly enhanced removal rates for degrading SMP compared with other previous photocatalysts. This finding highlights the significant innovation and validity of the DBHP for degrading SMP, further emphasizing that this study is significant in the field of the research and development of the visible-light-responsive photocatalysts that possess high photocatalytic activity for degrading organic pollutants.

## 2. Results

### 2.1. Characterization of Photocatalysts

#### 2.1.1. X-Ray Diffraction (XRD) Pattern Analysis

**Figure 1:**
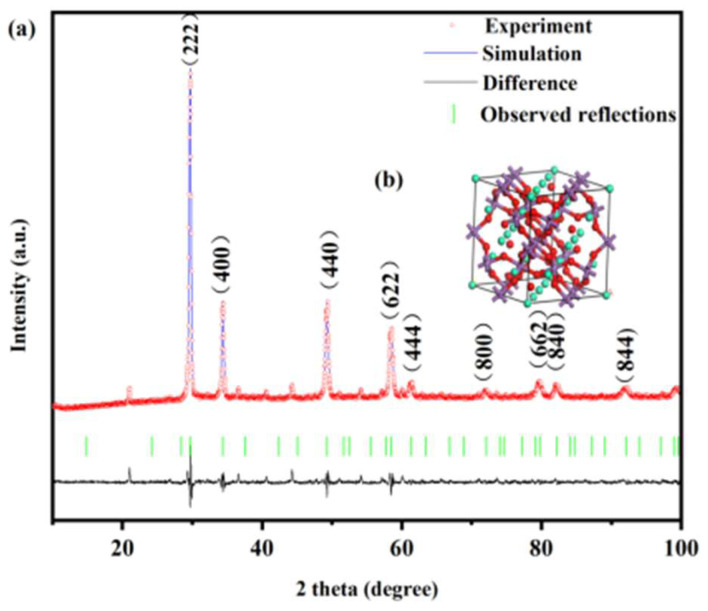
(**a**) XRD pattern of Dy_2_TmSbO_7_; (**b**) atomic structure schematic diagram (green atom: Dy; purple atom: Tm or Sb; red atom: O) of Dy_2_TmSbO_7_.

**Figure 2:**
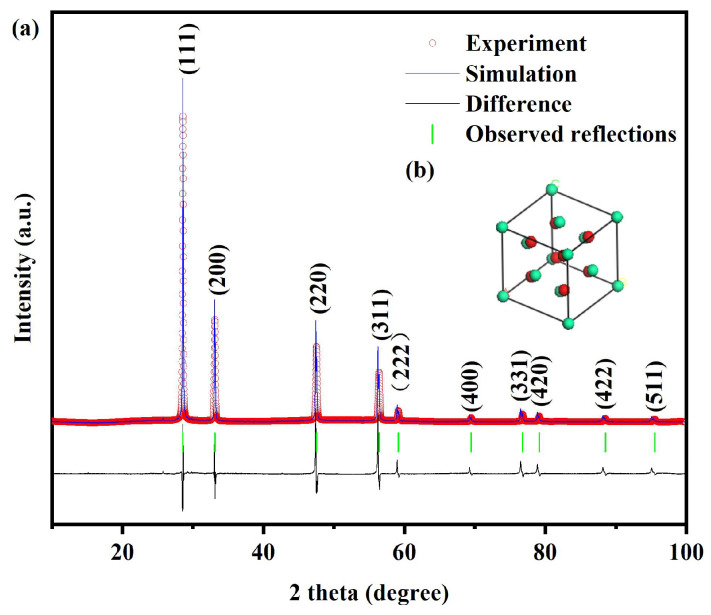
(**a**) XRD pattern of BiHoO_3_; (**b**) atomic structure schematic diagram (green atom: Bi or Ho; red atom: O) of BiHoO_3_.

**Figure 3:**
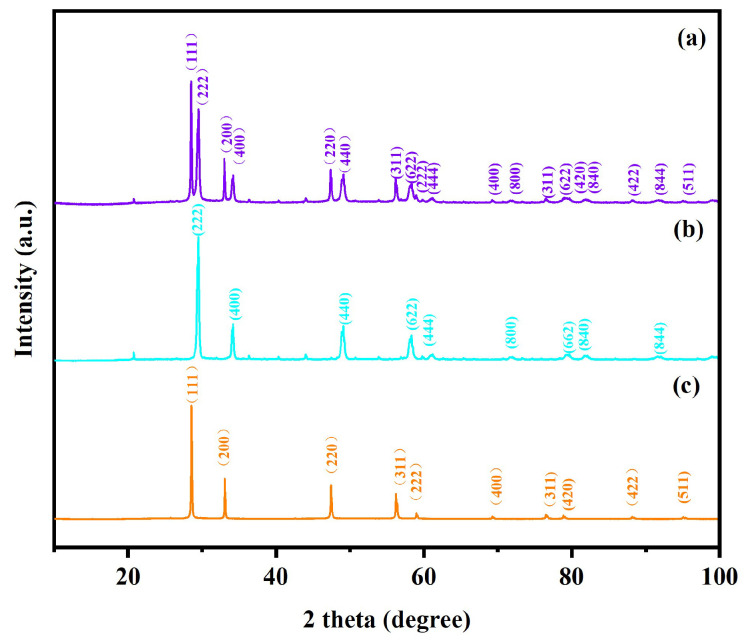
(**a**) XRD pattern of DBHP; (**b**) XRD pattern of Dy_2_TmSbO_7_; (**c**) XRD pattern of BiHoO_3_.

Figure 1a illustrates the XRD pattern and the Rietveld refinement results of Dy_2_TmSbO_7_. Figure 1b displays the atomic structure schematic diagram of Dy_2_TmSbO_7_. Figure 2a shows the XRD pattern and the Rietveld refinement results of the BiHoO_3_. Figure 2b exhibits the atomic structure schematic diagram of the BiHoO_3_. Both Dy_2_TmSbO_7_ and BiHoO_3_ were synthesized via the solid-state calcination method. Figure 3a displays the XRD pattern of the as-fabricated DBHP specimen. Figure 3b exhibits the XRD pattern of the as-fabricated Dy_2_TmSbO_7_ specimen. Figure 3c illustrates the XRD pattern of the as-fabricated BiHoO_3_ specimen. The Materials Studio software 2020 was applied to obtain the quantitative XRD data of the prepared photocatalysts in accordance with the Rietveld analysis results. As revealed in Figure 1a and Figure 2a, the results show that the Dy_2_TmSbO_7_ or the BiHoO_3_ was a single phase; meanwhile, the lattice parameter of the Dy_2_TmSbO_7_ was 10.52419 Å; moreover, the lattice parameter of the BiHoO_3_ was 5.42365 Å. As revealed in Figure 1a, the Rietveld refinement results of the Dy_2_TmSbO_7_ produced a deviation of 3.65%. Correspondingly, as revealed in Figure 2a, the Rietveld refinement results of the BiHoO_3_ produced a deviation of 9.69%; moreover, it could be observed in Figure 1a and Figure 2a that the experimental peak positions and the theoretical peak positions for Dy_2_TmSbO_7_ and BiHoO_3_ exhibited an exact one-to-one correspondence. Simultaneously, the profiles of Dy_2_TmSbO_7_ and BiHoO_3_ closely matched the shapes of Dy_2_TmSbO_7_ and BiHoO_3_ [51,52,53], indicating that a good consistency was realized between the experimental intensity and the theoretical intensity; therefore, the Dy_2_TmSbO_7_ was verified to have the pyrochlore-type crystal structure; concurrently, the BiHoO_3_ was verified to possess the fluorite structure [54]. Ultimately, the space group for the photocatalyst was Fd3m, and the space group for BiHoO_3_ was Fm3m. The DBHP was fabricated via the ultrasound-assisted solvothermal method [55,56]. It can be found in Figure 3a that the significant peaks which were observed in the XRD pattern of DBHP were congruous with those of Dy_2_TmSbO_7_ and those of BiHoO_3_, thereby the DBHP was synthesized with success.

According to Figure 1b and Figure 2b, the structures of Dy_2_TmSbO_7_ and BiHoO_3_ were constructed based on the corresponding space group, crystal system, lattice constant, atomic coordinates, and structural parameters. A comprehensive analysis result for the atomic coordinates and the structural parameters of Dy_2_TmSbO_7_ is given in the Supplementary Table S1. A comprehensive analysis result for the atomic coordinates and the structural parameters of the BiHoO_3_ is given in the Supplementary Table S2. In accordance with Figure 1b and Figure 2b and Tables S1 and S2, the above analytical results strongly verify the configurable stability of the Dy_2_TmSbO_7_ sample and the BiHoO_3_ sample. Simultaneously, the above analytical results also highlight the applied potential of the Dy_2_TmSbO_7_ sample and the BiHoO_3_ sample for the higher photocatalytic activity by purifying the polluted industrial wastewater.

The Dy_2_TmSbO_7_ compound contained two different A-O bonds: the longer bond, which was denoted as Dy-O (1), possessed a bond length of 2.279 Å; meanwhile, the shorter bond, which was denoted as Tm-O (2), had a bond length of 2.021 Å. The differentia, which stemmed from the bond lengths, was predicted to cause the distortion of the MO_6_ octahedra (M = Tm^3+^ and Sb^5+^); consequently, the recombination rate of the photogenerated electrons and the photogenerated holes was reduced. Consequently, the photocatalytic activity of the Dy_2_TmSbO_7_ photocatalyst was enhanced. The measured M-O-M bond angle was 134.020°. Obviously, previous studies have shown that a bond angle that was near to 180° could promote the mobility rate of photogenerated electrons and the photogenerated holes and the photocatalytic activity of the photocatalyst; accordingly, the larger M-O-M angle of 134.020° that originated from Dy_2_TmSbO_7_ further improved the photocatalytic activity of Dy_2_TmSbO_7_.

#### 2.1.2. Fourier Transform Infrared (FTIR) Spectrum Analysis

**Figure 4:**
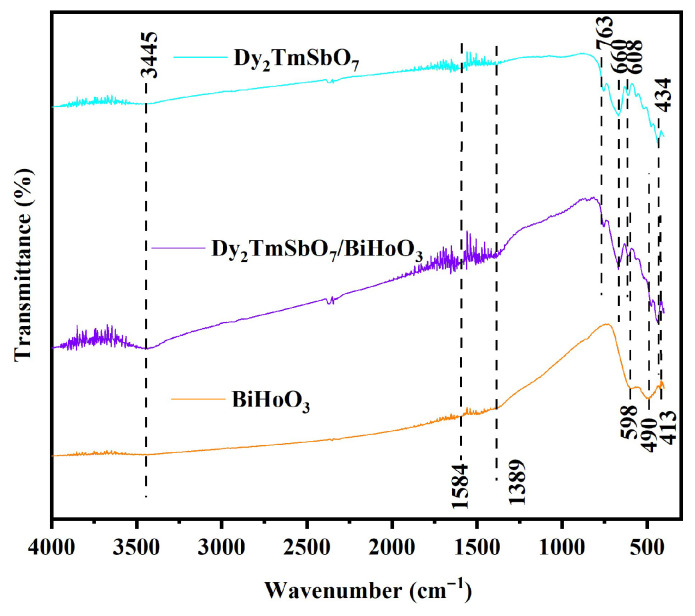
The FTIR spectra of Dy_2_TmSbO_7_, BiHoO_3_, and DBHP.

Figure 4 exhibits the Fourier transform infrared (FTIR) spectra of Dy_2_TmSbO_7_, BiHoO_3_, and DBHP. Figure 4 indicates the characterization of the functional groups that derived from the prepared samples that were detected using an FTIR spectrometer. It can be found from Figure 4 that the FTIR spectrum of the DBHP nanocomposite covered all the characteristic peaks that were observed in the Dy_2_TmSbO_7_ component and the BiHoO_3_ component. As revealed in Figure 4, in accordance with the FTIR spectrum of DBHP or BiHoO_3_, a unique stretching oscillation of the Bi-O bond was observed at a wavenumber of 413 cm^−1^ [57]. At one time, the bending oscillation of the Ho-O bond was checked at 598 cm^−1^ [58]. Moreover, a band which correlated to the Dy-O bond appeared at 434 cm^−1^ [59]. Additionally, the stretching oscillation of the Tm-O bond was attributed to the band that was located at 660 cm^−1^ [60]. Simultaneously, the stretching oscillation, which was related to the Sb-O-Sb bond, could be observed at 608 cm^−1^ and 763 cm^−1^ [61]. Furthermore, the FTIR spectra which originated from Figure 4 indicates that the spectral absorption band’s center was located at 3445 cm^−1^, which might be caused by the valence oscillation of water molecules [62]. Another absorption band, which was located at 1584 cm^−1^, was identified as the characteristic stretching of the H-O-H bond; in addition, the peak, which was observed at 1389 cm^−1^, was attributed to the O-H vibrational mode of water molecules, which were adsorbed on the sample surface [63,64]; nevertheless, the prepared photocatalysts were specifically intended for degrading SMP contained in water. Therefore, the presence of a small amount of the adsorbed water that existed on the surface of the prepared photocatalysts did not affect the validity of our research findings. In general, the above findings strongly verify the existence and stability of the heterostructure photocatalyst which was composed of Dy_2_TmSbO_7_ and BiHoO_3_, providing significant views for the further understanding and application of Dy_2_TmSbO_7_, BiHoO_3_, and DBHP. Finally, we have placed the data table of various chemical bonds and their corresponding peaks which are derived from the FTIR spectra of the Dy_2_TmSbO_7_/BiHoO_3_ heterostructure photocatalyst, Dy_2_TmSbO_7_, and BiHoO_3_; as a result, the above data information can be found in Table S3 of the Supplementary Materials.

#### 2.1.3. Raman Spectrum Analysis

**Figure 5:**
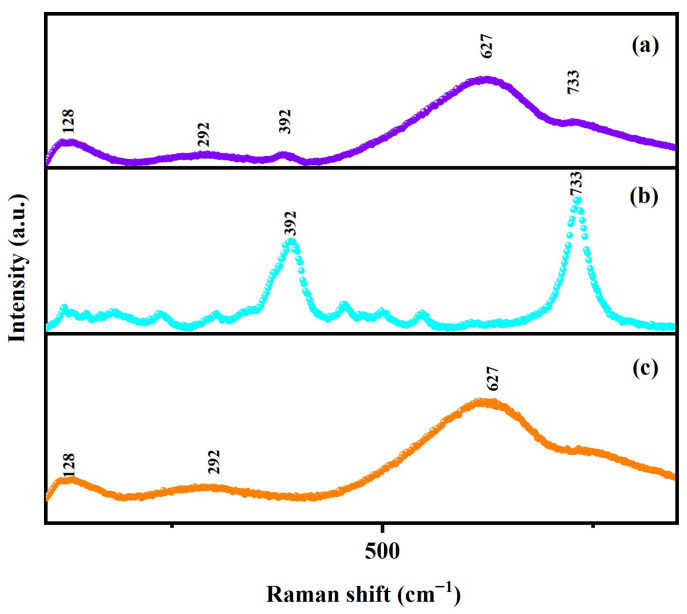
(**a**) Raman spectrum of DBHP; (**b**) Raman spectrum of Dy_2_TmSbO_7_; (**c**) Raman spectrum of BiHoO_3_.

In order to attain the interaction rules derived from the various chemical bonds of DBHP, Dy_2_TmSbO_7_, and BiHoO_3_, the Raman spectra were acquired using a Raman spectrometer. Figure 5a shows the Raman spectrum of DBHP, which was prepared using the ultrasonic-assisted solvothermal method. Figure 5b exhibits the Raman spectrum of Dy_2_TmSbO_7_, which was prepared using the solid-state calcination method. Figure 5c displays the Raman spectrum of BiHoO_3_, which was prepared using the solid-state calcination method. As revealed in Figure 5a–c, significant patterns can be observed in the Raman spectra of DBHP, Dy_2_TmSbO_7_, and BiHoO_3_. The peak located at 733 cm^−1^ is attributed to Dy-O bonds [65]. The strongest peak located at 392 cm^−1^ is correlated to the Ag mode of the Tm-O bonds or the combined mode of the Ag mode and Fg mode [66]. The peak located at 627 cm^−1^ could be ascribed to the stretching oscillation of the Bi-O bonds that originated from the BiO_6_ octahedron [67]. Moreover, the stretched vibration of the Ho-O bonds are observed at 292 cm^−1^ [68]. These observed peaks confirmed the existence of the pure phase of the Dy_2_TmSbO_7_ photocatalyst and the pure phase of the BiHoO_3_ photocatalyst. Simultaneously, the abovementioned observed peaks are consistent with the XRD pattern results that were gained from the Dy_2_TmSbO_7_ and BiHoO_3_. The Raman spectrum of the DBHP exhibited obvious peak that were located at 292 cm^−1^, 392 cm^−1^, 627 cm^−1^, or 733 cm^−1^, indicating that the inherent unique traits of Dy_2_TmSbO_7_ and BiHoO_3_ were integrated. Thus, the above observations further confirm the successful preparation of DBHP. In addition, we have placed the data table of various chemical bonds and their corresponding peaks derived from the Raman spectra of the Dy_2_TmSbO_7_/BiHoO_3_ heterostructure photocatalyst, Dy_2_TmSbO_7_, and BiHoO_3_; as a result, the above data information can be found in Table S4 of the Supplementary Materials.

### 2.2. Property Characterization of Dy_2_TmSbO_7_/BiHoO_3_ Heterojunction Photocatalyst

#### 2.2.1. Morphology Image and Elemental Distribution Analysis

**Figure 6:**
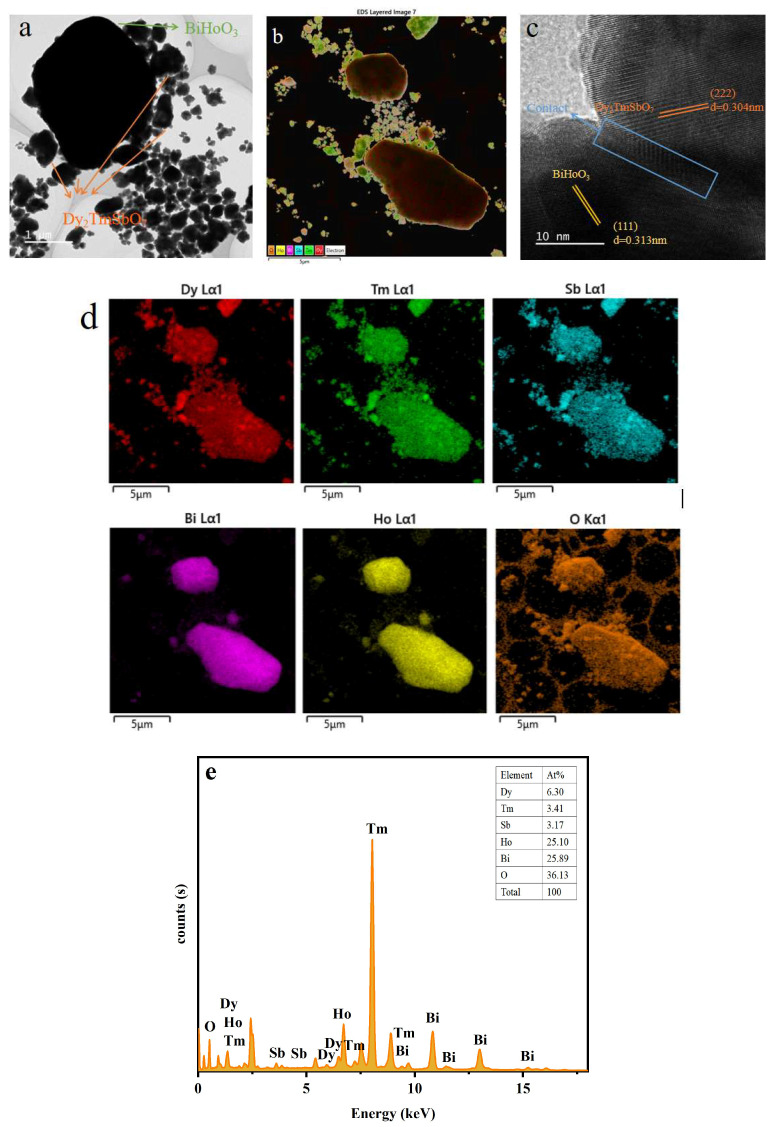
(**a**) TEM image of DBHP; (**b**) layered EDS composition distribution region of DBHP particle; (**c**) HRTEM image of DBHP; (**d**) EDS elemental scanning mapping images of DBHP; (**e**) EDS composition content image of DBHP.

The surface trait, lattice fringes, elemental composition content, and elemental composition distribution of the synthetic DBHP were analyzed sequentially using the transmission electron microscopy (TEM), high-resolution transmission electron microscopy (HRTEM), and energy-dispersive X-ray spectroscopy (EDS). Figure 6a exhibits the TEM morphology image of the DBHP. Figure 6b displays the layered EDS composition distribution region of the DBHP particle. Figure 6c exhibits an HRTEM image of the DBHP. Figure 6d illustrates EDS elemental scanning mapping images of the DBHP. Figure 6e indicates an EDS composition content distribution image of the DBHP. It can be found from Figure 6a that the presence of Dy_2_TmSbO_7_ nanoparticles and BiHoO_3_ nanoparticles ascribed to the DBHP are depicted. In Figure 6b, a TEM morphological image of the EDS measurement area remained with the DBHP and is exhibited. In Figure 6c, an HRTEM image of the interface is situated between the Dy_2_TmSbO_7_ nanoparticles and the BiHoO_3_ nanoparticles which were signed in Figure 6a is displayed. Obviously, the interface which is situated between the Dy_2_TmSbO_7_ nanoparticle and the BiHoO_3_ nanoparticle is clearly observed; meanwhile, two distinct lattice fringes were discovered and calculated for obtaining the indices of the crystallographic plane [69,70]. The calculated interplanar distance of the Dy_2_TmSbO_7_ was 0.304 nm, which is consistent with the crystal face index of (222) for Dy_2_TmSbO_7_; meanwhile, the calculated interplanar distance of BiHoO_3_ was 0.313 nm, which is consistent with the crystal face index of (111) for BiHoO_3_. In Figure 6d, the presence of the Dy element, Tm element, Sb element, Bi element, Ho element, and O element, which stemmed from the DBHP sample, was confirmed by the EDS elemental mapping analysis result. When the luminescent regions that were related to the Dy element, Ho element, Bi element, Tm element, and Sb element were investigated carefully, it could be inferred that BiHoO_3_ corresponded to the larger particles, contrarily, Dy_2_TmSbO_7_ corresponded to the smaller particles. Furthermore, in accordance with the EDS composition content distribution image revealed in Figure 6e, the atomic ratio of Dy element, Tm element, Sb element, Bi element, Ho element, and O element was approximately 630:341:317:2589:2510:3613. In conclusion, the successful preparation of DBHP was strongly confirmed.

#### 2.2.2. X-Ray Photoelectron Spectrometer Spectrum Analysis

**Figure 7:**
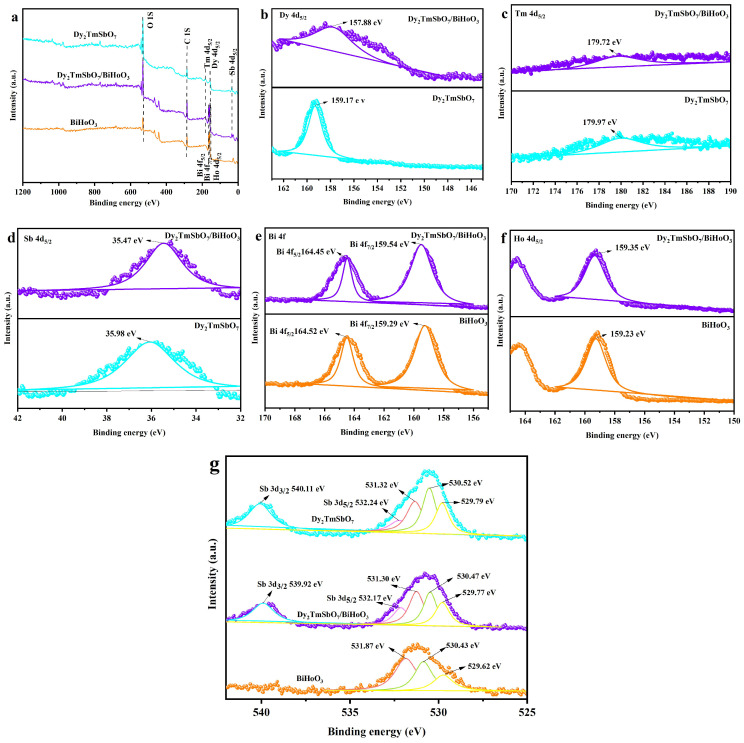
(**a**) Full XPS spectra of DBHP, Dy_2_TmSbO_7_, and BiHoO_3_; (**b**) XPS spectra of Dy 4d for DBHP and Dy_2_TmSbO_7_; (**c**) XPS spectra of Tm 4d for DBHP and Dy_2_TmSbO_7_; (**d**) XPS spectra of Sb 4d for DBHP and Dy_2_TmSbO_7_; (**e**) XPS spectra of Bi 4f for DBHP and BiHoO_3_; (**f**) XPS spectra of Ho 4d for DBHP and BiHoO_3_; (**g**) XPS spectra of O 1s for DBHP, Dy_2_TmSbO_7_, and BiHoO_3_; (**g**) XPS spectra of Sb 3d for DBHP and Dy_2_TmSbO_7_.

Figure 7a shows the full-scan X-ray photoelectron spectrometer (XPS) spectrum of DBHP, Dy_2_TmSbO_7_, or BiHoO_3_. Figure 7b displays the XPS spectra of Dy 4d for DBHP and Dy_2_TmSbO_7_. Figure 7c illustrates the XPS spectra of Tm 4d for DBHP and Dy_2_TmSbO_7_. Figure 7d exhibits the XPS spectra of Sb 4d for DBHP and Dy_2_TmSbO_7_. Figure 7e indicates the XPS spectra of Bi 4f for DBHP and BiHoO_3_. Figure 7f presents the XPS spectra of Ho 4d for DBHP and BiHoO_3_. Figure 7g represents the XPS spectra of O 1s for DBHP, Dy_2_TmSbO_7_, and BiHoO_3_. Figure 7g also exhibits the XPS spectra of Sb 3d for DBHP and Dy_2_TmSbO_7_. The XPS spectrum was applicable to investigating the surface chemical composition distribution, the surface chemical composition content, and the elemental chemical valence of DBHP, Dy_2_TmSbO_7_, and BiHoO_3_. The measured spectra which are represented from Figure 7a to Figure 7g showed that the Dy element, Tm element, Sb element, Bi element, Ho element, and O element were detected in DBHP; simultaneously, the carbon peak served as a calibration reference in DBHP [71]. The above result indicates that Dy_2_TmSbO_7_ and BiHoO_3_ were successfully incorporated into DBHP. The spectral peak corresponding to Dy 4d_5/2_, Tm 4d_5/2_, Sb 4d_5/2_, Bi 4f_7/2_, Bi 4f_5/2_, or Ho 4d_5/2_, which derived from DBHP, Dy_2_TmSbO_7_, or BiHoO_3_, can be found in Figure 7b–e or Figure 7f. In Figure 7b, the Dy 4d_5/2_ peak of DBHP shifted to a lower binding energy; simultaneously, the center of the Dy 4d_5/2_ peak was located at 157.88 eV. In Figure 7c, the Tm 4d_5/2_ peak of DBHP shifted to a lower binding energy; correspondingly, the center of the Tm 4d_5/2_ peak was located at 179.72 eV. In Figure 7d, the Sb 4d_5/2_ peak in DBHP shifted to a lower binding energy; concomitantly, the center of the Sb 4d_5/2_ peak was located at 35.47 eV. Figure 7e indicated that the Bi 4f_7/2_ peak and the Bi 4f_5/2_ peak which originated from DBHP shifted to higher binding energies, and the peak which was located at 159.54 eV or 164.45 eV in the Bi 4f spectrum of DBHP was assigned to the Bi 4f_7/2_ state of Bi^3+^ or the Bi 4f_5/2_ state of Bi^3+^, which was 159.54 eV or 164.45 eV; as a result, the above conclusion was supported by a spin-orbit splitting value of 4.91 eV [72]. As revealed in Figure 7f, the Ho 4d_5/2_ peak which stemmed from DBHP shifted to a higher binding energy; incidentally, the center of the Ho 4d_5/2_ peak was located at 179.72 eV. The deconvoluted O 1s spectra of DBHP, Dy_2_TmSbO_7_, and BiHoO_3_ are shown in Figure 7g exhibiting distinct peaks at 529.77 eV, 529.79 eV, or 529.62 eV, indicating that lattice oxygen was presented [73]; in addition, peaks which were observed at 530.47 eV, 530.52 eV, or 530.83 eV indicate the presence of the hydroxyl group; finally, the peaks at 531.39 eV, 531.37 eV, or 531.80 eV indicate the presence of the oxygen vacancy. As revealed in Figure 7g, the binding energies of the Sb 3d_5/2_ peak and the Sb 3d_3/2_ peak which derived from DBHP were lower than those which stemmed from Dy_2_TmSbO_7_. Moreover, in DBHP, the center of the Sb 3d_5/2_ peak or the Sb 3d_3/2_ peak was located at 532.17 eV or 539.92 eV, respectively; therefore, the above result indicate that Sb was in the +5 oxidation state [74]. The binding energy of Bi 4f or Ho 4d which derived from the DBHP was higher than that from BiHoO_3_; meanwhile, the binding energy of Dy 4d, Tm 4d, or Sb 3d which originated from the DBHP was lower than that from Dy_2_TmSbO_7_. The above results could be explained by the lower electron density of Bi or Ho which derived from the DBHP and the higher electron density of Dy, Tm, or Sb which stemmed from the DBHP [75]. In accordance with Figure 7a–g, it can be found that the molar ratio of the Dy element, Tm element, Sb element, Bi element, Ho element, and O element was around 1163:617:636:622:631:6420, which is consistent with the EDS composition content distribution result deriving from the atomic ratio of the Dy element, Tm element, Sb element, Bi element, Ho element, and O element stemming from DBHP. The peak analysis results of the XPS spectrum for DBHP, Dy_2_TmSbO_7_, and BiHoO_3_ did not find the existence of any other phases. In conclusion, these XPS spectrum observations confirmed the powerful chemical interaction between Dy_2_TmSbO_7_ and BiHoO_3_. Concurrently, these XPS spectrum observations also proved the existence of the heterostructure for DBHP.

### 2.3. Optical Characteristics of the Prepared Photocatalysts

#### 2.3.1. UV-Vis Absorption Spectra

**Figure 8:**
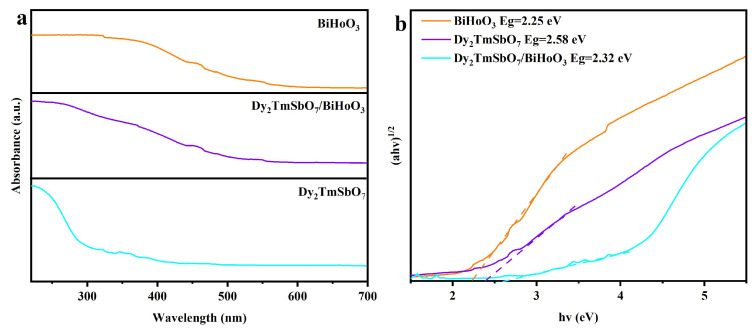
(**a**) UV-Vis absorption spectra of DBHP, Dy_2_TmSbO_7_, and BiHoO_3_; (**b**) corresponding plots of (*αhν*)^1/2^ and *hν* for DBHP, Dy_2_TmSbO_7_, and BiHoO_3_.

Figure 8a displays the ultraviolet and visible absorption spectra of DBHP, Dy_2_TmSbO_7_, and BiHoO_3_. Figure 8a shows that the intrinsic transition absorption edge of Dy_2_TmSbO_7_ started at a wavelength of approximately 460 nm; simultaneously, the inherent transition absorption edge of BiHoO_3_ started at a wavelength of 590 nm; moreover, the intrinsic transition absorption edge of the DBHP started at a wavelength of 560 nm. Obviously, the inchoative wavelength of the intrinsic transition absorption edge for the DBHP shifted toward a longer wavelength direction compared with that for Dy_2_TmSbO_7_. The above results indicate that the visible light absorption capacity of DBHP was superior to that of Dy_2_TmSbO_7_.

Figure 8b shows the corresponding plot of (*αhν*)^1/2^ versus *hν* for DBHP, Dy_2_TmSbO_7_, or BiHoO_3_. The optical absorption characteristic, which is displayed near the band edge of the crystalline semiconductor oxide catalyst, conformed to Equation (1); according to Equation (1), the band gap energy of DBHP, Dy_2_TmSbO_7_, or BiHoO_3_ can be calculated; subsequently, the visible light absorption capacity of DBHP, Dy_2_TmSbO_7_, or BiHoO_3_ can be preliminarily determined [76,77].

(1) $$\alpha h\nu =A(h\nu -Eg{)}^{n}$$

According to Equation (1), *A*, *α*, *h*, *Eg* or *v* represent the scalable numerical constant, absorption modulus, Planck constant, band gap width, or incident light frequency. The parameter “*n*” was used for characterizing the type of electronic transition after optical excitation; correspondingly, the “*n*” value of 1/2 represents direct transition; meanwhile, the “*n*” value of 2 represents indirect transition. According to the plots shown in Figure 8b, the measured band gap width value of 2.58 eV for Dy_2_TmSbO_7_ was obtained; concurrently, the measured band gap width value of 2.25 eV for BiHoO_3_ was gained. Ultimately, the measured band gap width value of 2.32 eV for DBHP was achieved, indicating DBHP possessed a narrower band gap width compared with Dy_2_TmSbO_7_. In conclusion, the above experimental results meant that DBHP, Dy_2_TmSbO_7_, and BiHoO_3_ had visible light response characteristics and high absorption capacity for visible light.

#### 2.3.2. Photoluminescence Spectrum

**Figure 9:**
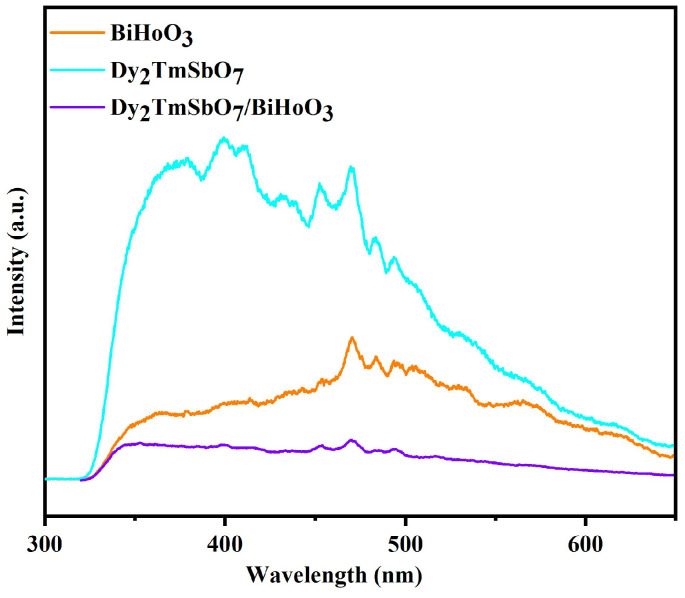
**The** PL spectra of DBHP, Dy_2_TmSbO_7_, and BiHoO_3_.

Figure 9 shows photoluminescence (PL) spectrum of DBHP, Dy_2_TmSbO_7_, or BiHoO_3_. In accordance with Figure 9, the recombination rate of the photogenerated electrons and the photogenerated holes can be obtained. The lower the PL intensity, the lower the restructuring rate of the photogenerated electrons and the photogenerated holes, and the photocatalytic activity of the DBHP might be enhanced [78,79]. As revealed in Figure 9, a broad emission peak which derived from the PL spectrum of the DBHP, Dy_2_TmSbO_7_, or BiHoO_3_ was observed around 470 nm. Obviously, the broad emission peak which centered at 470 nm was a common characteristic of many wide bandgap semiconductors and metal oxides; moreover, the broad emission peak which centered at 470 nm was typically attributed to the defect related to the luminescence. Under the condition of appropriate excitation, DBHP, Dy_2_TmSbO_7_, and BiHoO_3_ exhibited a broad emission band which centered at approximately 470 nm, which was mainly ascribed to the radiative recombination of defect states within the bandgap [80,81]. In such complex oxide semiconductors, these defects often included oxygen vacancies, which could form trap states below the conduction band minimum. The photogenerated electrons and the photogenerated holes could be trapped by these defect states and subsequently undergo radiative recombination; therefore, the observed sub bandgap emission was generated [82]. Simultaneously, BiHoO_3_ exhibited the highest PL intensity compared with DBHP or Dy_2_TmSbO_7_; contrarily, DBHP exhibited the lowest PL intensity compared with Dy_2_TmSbO_7_ or BiHoO_3_. Obviously, the above resultant trend indicates that the separation rate of the photogenerated electrons and the photogenerated holes which originated from the DBHP was accelerated compared with that from Dy_2_TmSbO_7_ or from BiHoO_3_: therefore, a higher photocatalytic activity of DBHP was realized.

#### 2.3.3. Time-Resolved Photoluminescence Spectra

**Figure 10:**
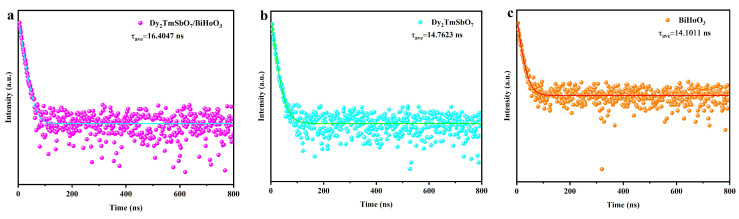
(**a**) TRPL spectrum of DBHP; (**b**) TRPL spectrum of Dy_2_TmSbO_7_; (**c**) TRPL spectrum of the BiHoO_3_.

Figure 10a shows the time-resolved photoluminescence (TRPL) spectrum of DBHP. Figure 10b displays the TRPL spectrum of Dy_2_TmSbO_7_. Figure 10c exhibits the TRPL spectrum of BiHoO_3_. According to the Equation (2), Figure 10a–c, the average lifetime of the photogenerated electrons that were derived from DBHP, Dy_2_TmSbO_7_, or BiHoO_3_ was calculated [83,84].

(2) $$\tau_{ave}=\left(A_{1}\tau_{1}^{2}+A_{2}\tau_{2}^{2}\right)/\left(A_{1}\tau_{1}+A_{2}\tau_{2}\right)$$

The average lifetime that originated from Dy_2_TmSbO_7_ was 14.7623 ± 0.0829 ns; meanwhile, the average lifetime that originated from BiHoO_3_ was 14.1011 ± 0.0395 ns; ultimately, the average lifetime that originated from DBHP was 16.4047 ± 0.0283 ns. DBHP showed a significantly prolonged lifetime of photogenerated electrons compared with Dy_2_TmSbO_7_ or BiHoO_3_. The shorter average lifetime of photogenerated electrons that derived from Dy_2_TmSbO_7_ or BiHoO_3_ indicates the rapid recombination efficiency of the photogenerated electrons and the photogenerated holes. Contrarily, the longer lifetime of photogenerated electrons that originated from DBHP was attributed to the improved separation efficiency of photogenerated electrons and the photogenerated holes within DBHP. The TRPL spectra results that were obtained as shown in Figure 10a–c are consistent with the above PL spectra results.

#### 2.3.4. Photocurrent Density Variation Analysis

**Figure 11:**
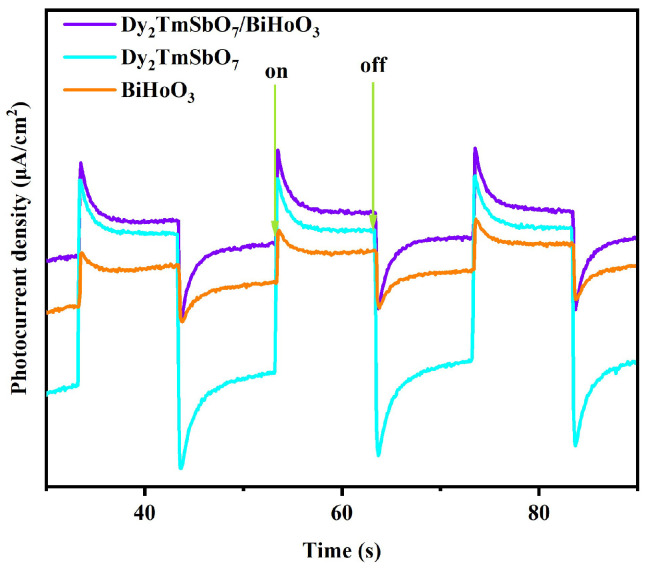
Influence of the photocurrent density on the visible light irradiation time with the Dy_2_TmSbO_7_, BiHoO_3_, or DBHP as the photoelectrode under visible light irradiation.

Figure 11 shows the influence of the photocurrent density on the visible light irradiation time with Dy_2_TmSbO_7_, BiHoO_3_, or DBHP as a photoelectrode under visible light irradiation. It is well known that the photocurrent response density directly affects the photogenerated electrons and the photogenerated holes density, thus, a higher photocurrent density corresponded to higher photogenerated electron and photogenerated hole densities [85]. In accordance with Figure 11, DBHP exhibited a higher photocurrent density compared with the single Dy_2_TmSbO_7_ sample or the single BiHoO_3_ sample, indicating that the integration of Dy_2_TmSbO_7_ and BiHoO_3_ might promote the efficient separation of the photogenerated electrons and the photogenerated holes. The above photocurrent density observation which derived from Figure 11 was in keeping with the enhancive separation efficiency of the photogenerated electrons and the photogenerated holes that stemmed from the PL and TRPL spectra of the effects.

#### 2.3.5. Nyquist Plot of the Electrochemical Impedance Spectroscopy

**Figure 12:**
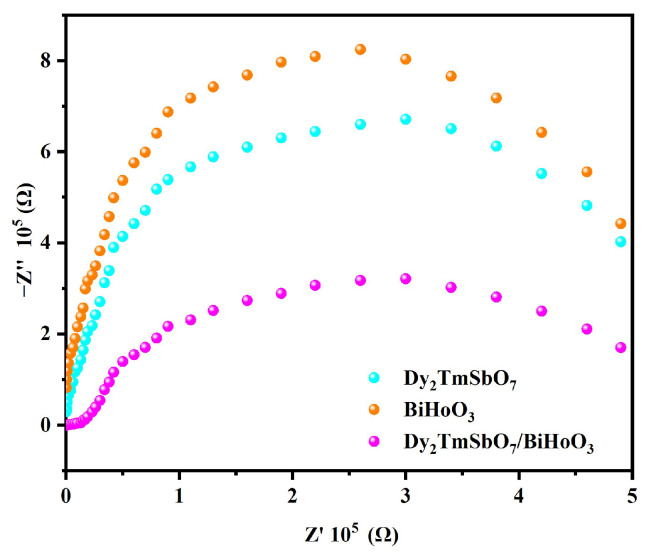
EIS plots of Dy_2_TmSbO_7_, BiHoO_3_, and DBHP.

Figure 12 shows a Nyquist plot of the electrochemical impedance spectroscopy (EIS) using DBHP, Dy_2_TmSbO_7_, or BiHoO_3_. The Nyquist plots which were shown in Figure 12 were utilized for further studying the separation efficiency of photogenerated electrons and the photogenerated holes. In the Nyquist plot displayed in Figure 12, the smaller the radius was, the smaller the charge transfer resistance generally was. As revealed in Figure 12, DBHP exhibited a smaller radius compared with Dy_2_TmSbO_7_ or BiHoO_3_, indicating that DBHP possessed a higher separation efficiency of the photogenerated electrons and the photogenerated holes compared with Dy_2_TmSbO_7_ or BiHoO_3_. The above results obtained in Figure 12 verify the PL results and the TRPL spectra results, as well as the transient photocurrent response results. Ultimately, the above results confirm that the heterojunction structure which originated from Dy_2_TmSbO_7_ and BiHoO_3_ significantly accelerated the separation efficiency of the photogenerated electrons and the photogenerated holes, thereby the lifetime of the photogenerated electrons and the photogenerated holes was protracted.

#### 2.3.6. The N_2_ Adsorption-Desorption Isotherm

The N_2_ adsorption–desorption isotherms of DBHP, Dy_2_TmSbO_7_, and BiHoO_3_ are exhibited in Figure S1. The Brunauer–Emmett–Teller specific surface areas of Dy_2_TmSbO_7_, BiHoO_3_ and DBHP were determined to be 2.57 ± 0.27 m^2^/g, 2.18 ± 0.13 m^2^/g, and 2.34 ± 0.09 m^2^/g. Notably, the DBHP exhibited a larger specific surface area than BiHoO_3_. A larger specific surface area implies a greater number of active sites that are available for photodegradation reactions; therefore, the DBHP can improve the photocatalytic activity because of larger specific surface area.

### 2.4. Examination of Photocatalytic Efficiency

**Figure 13:**
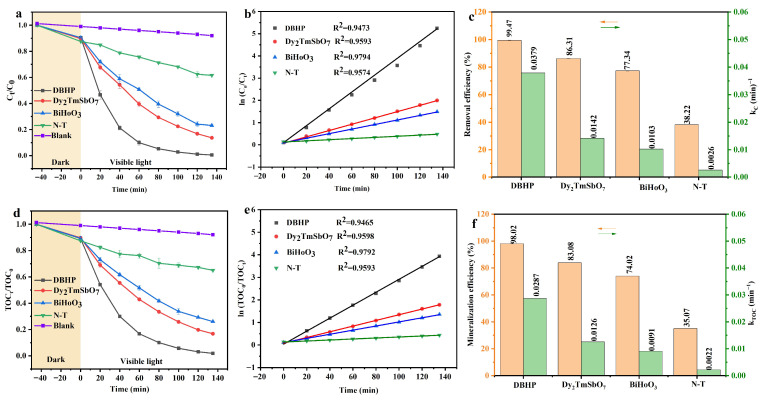
(**a**) The PADA curve with the DBHP, Dy_2_TmSbO_7_, BiHoO_3_, or N-T; (**b**) kinetic curve with DBHP, Dy_2_TmSbO_7_, BiHoO_3_, or N-T; (**c**) removal rate and kinetic constants for degrading SMP with DBHP, Dy_2_TmSbO_7_, BiHoO_3_, or N-T; (**d**) mineralization efficiency curves with DBHP, Dy_2_TmSbO_7_, BiHoO_3_, or N-T; (**e**) kinetic curve with DBHP, Dy_2_TmSbO_7_, BiHoO_3_, or N-T; (**f**) mineralization efficiency and kinetic constants for removing the TOC concn with DBHP, Dy_2_TmSbO_7_, BiHoO_3_, or N-T.

Figure 13a shows the mutation curve that is engendered by the effect of the value of *C_t_*/*C*_0_ on the visible light irradiation time for the photocatalytic degradation (PADA) of SMP using DBHP, Dy_2_TmSbO_7_, BiHoO_3_, or nitrogen-doped TiO_2_ (N-T). *C*_0_ which appears in Figure 13a represents the original concn of SMP; concurrently, *C_t_* which appears in Figure 13a represents the concn of SMP at a transient visible light irradiation time. The reason that we selected N-T as the contradistinctive catalyst was primarily due to market feedback for mass-produced N-T, which possesses an inexpensive price and definite visible light responsive characteristic. SMP solution was initially stirred in darkness for 45 min to achieve equilibrium between the adsorption state and the desorption state. Figure 13a indicates that the prepared catalyst, such as DBHP, Dy_2_TmSbO_7_, or BiHoO_3_, achieved a significant degradation efficiency of SMP, and DBHP exhibited a significantly superior performance compared with Dy_2_TmSbO_7_, BiHoO_3_, or N-T. In contrast, the control experiment, which was conducted only by photolysis without adding the catalyst, showed that there was no obvious change in the SMP concn. The above result indicates that the photocatalytic activity of the synthesized catalyst, such as DBHP, Dy_2_TmSbO_7_, or BiHoO_3_, possessed a definite effect on the photodegradation efficiency. Figure 13b shows the corresponding effect of ln(*C*_0_/*C_t_*) on the visible light irradiation time. Figure 13b shows that the above result conformed to the first-order kinetic model. Generally, if R^2^ was greater than 0.95, the first-order kinetic model could be applied. We found that the R^2^ value was close to 0.99 using DBHP, Dy_2_TmSbO_7_, or BiHoO_3_ under visible light irradiation in our study [86,87]. Meanwhile, the kinetic constant which was derived from the effect of the SMP concn on the visible light irradiation time in the PADA of the SMP could be calculated.

In order to clarify the enhanced photocatalytic activity of the prepared photocatalysts under visible light irradiation, Figure 13c exhibits the photodegradation efficiency (PRE) of SMP and the kinetic constants which are caused by the effect of the ln(*C*_0_/*C_t_*) value on the visible light irradiation time using DBHP, Dy_2_TmSbO_7_, BiHoO_3_, or N-T. The kinetic constant which was obtained according to the effect of ln(*C*_0_/*C_t_*) on the visible light irradiation time was calculated using the standard formula of ln(*C*_0_/*C_t_*) = *k_C_t*. In accordance with Figure 13c, the PRE of SMP after visible light irradiation of 135 min with DBHP was 99.47%. Specifically, the PRE of SMP after visible light irradiation of 135 min with DBHP was approximately 1.15 times that of using the original Dy_2_TmSbO_7_, 1.29 times that of using the original BiHoO_3_ or 2.60 times that of using the original N-T. In addition, with DBHP, Dy_2_TmSbO_7_, BiHoO_3_, or N-T under visible light irradiation in the PADA process, the first-order kinetic constant, *k_C_*, was gained to be 0.0379 min^−1^, 0.0142 min^−1^, 0.0103 min^−1^, or 0.0026 min^−1^. Simultaneously, Figure 13c showed that the kinetic constant, *k*, value that was obtained with DBHP under visible light irradiation surpassed that of Dy_2_TmSbO_7_, BiHoO_3_, or N-T in the PADA process. The kinetic constant which was formed according to the effect of the ln(*C*_0_/*C_t_*) value on the visible light irradiation time with DBHP was approximately 2.67 times, 3.01 times, or 14.56 times that of Dy_2_TmSbO_7_, BiHoO_3_, or N-T. In addition, the reaction rate for degrading SMP with DBHP, Dy_2_TmSbO_7_, BiHoO_3_, or N-T was 3.51 × 10^−9^ mol·L^−1^·s^−1^, 3.00 × 10^−9^ mol·L^−1^·s^−1^, 2.62 × 10^−9^ mol·L^−1^·s^−1^, or 1.01 × 10^−9^ mol/L·s^−1^ under visible light irradiation. The above experimental results displayed that the photocatalytic activity of the photocatalysts varied in the following order: DBHP > Dy_2_TmSbO_7_ > BiHoO_3_ > N-T. According to Equation (3), the calculated photon efficiency (PHEY) value for degrading SMP in the PADA process with DBHP, Dy_2_TmSbO_7_, BiHoO_3_, or N-T was 0.0737%, 0.0630%, 0.0550%, or 0.021% under visible light irradiation. The above results indicate that the value of the PHEY for degrading SMP with DBHP was the highest compared with that of using Dy_2_TmSbO_7_, BiHoO_3_, or N-T under visible light irradiation. PHEY was calculated using the following Equation (3):

(3) $$\phi =R/I_{0}$$

where ϕ is PHEY (%), R is the degradation rate of SMP (mol·L^−1^·s^−1^), and $I_{0}$ is the irradiation photon flux (Einstein L^−1^ s^−1^) [88,89,90,91].

Figure 13d illustrates the variation curve that is caused by the effect of the value of *TOC_t_*/*TOC*_0_ on the visible light irradiation time with DBHP, Dy_2_TmSbO_7_, BiHoO_3_, or nitrogen-doped TiO_2_ (N-T) for degrading SMP in the PADA process. *TOC_t_* refers to the TOC concentration at an instantaneous visible light irradiation time; *TOC*_0_ represents the initial TOC concentration. It can be observed from Figure 13d that the prepared catalyst, such as DBHP, Dy_2_TmSbO_7_, or BiHoO_3_, achieved desirable mineralization efficiency for removing the TOC concentration; simultaneously, DBHP exhibited excellent photocatalytic activity for degrading SMP compared with Dy_2_TmSbO_7_, BiHoO_3_, or N-T under visible light irradiation. Figure 13e depicts how ln(*TOC*_0_/*TOC_t_*) correlates with the visible light irradiation time throughout with DBHP, Dy_2_TmSbO_7_, BiHoO_3_, or N-T for degrading SMP in the PADA process. The data derived from Figure 13e follows the first-order kinetic model. Additionally, the kinetic constant that is associated with TOC concn changes over the visible light irradiation time during the PADA of SMP with these catalysts could be determined.

For the sake of the mineralization efficiency in the PADA of SMP with various catalysts, Figure 13f presents the mineralization efficiency for removing the TOC concentration and the kinetic constant after visible light irradiation of 135 min for degrading SMP in the PADA process with DBHP, Dy_2_TmSbO_7_, BiHoO_3_, or N-T. The kinetic constant which was determined based on the relationship between ln(*TOC*_0_/*TOC_t_*) and the visible light irradiation time using DBHP, Dy_2_TmSbO_7_, BiHoO_3_, or N-T was calculated with the standard formula of ln(*TOC*_0_/*TOC_t_*) = *k_TOC_*t. Figure 13f indicates that the TOC mineralization efficiency was 98.02% for DBHP following 135 min of visible light irradiation. Specifically, DBHP exhibited a TOC removal efficiency that was 1.18, 1.32, or 2.79 times higher than that achieved with the original Dy_2_TmSbO_7_, BiHoO_3_, or N-T after 135 min of visible light irradiation. The kinetic constant *k_TOC_* was 0.0287 min^−1^, 0.0126 min^−1^, 0.0091 min^−1^, or 0.0022 min^−1^ using DBHP, Dy_2_TmSbO_7_, BiHoO_3_, or N-T for degrading SMP under visible light irradiation. Figure 13f revealed that the kinetic constant *k_TOC_* value which was obtained in the PADA of SMP under visible light irradiation with DBHP was higher than that using Dy_2_TmSbO_7_, BiHoO_3_, or N-T. The kinetic constant which was formed in light of the effect of the ln(*TOC*_0_/*TOC_t_*) value on the visible light irradiation time with DBHP was approximately 2.78 times that using the original Dy_2_TmSbO_7_, 3.15 times that using the original BiHoO_3_, or 13.05 times that using the original N-T. Therefore, the obtained kinetic constant *k_TOC_* value with DBHP for degrading SMP in the PADA process under visible light irradiation was the highest among that using Dy_2_TmSbO_7_, BiHoO_3_, or N-T. Collectively, the above experimental data confirm the superior photocatalytic performance of DBHP over Dy_2_TmSbO_7_, BiHoO_3_, or N-T at degrading SMP under visible light irradiation. The three-dimensional fluorescence spectra of photocatalytic degradation of SMP with DBHP are provided in Figure S2 in the Supplementary Materials. As revealed in Figure S2, it can be observed that the fluorescence centers of SMP gradually weakened with increasing light irradiation time during the PADA process of SMP, indicating that the progressive degradation of SMP was realized and the intermediate products were formed during the PADA process of SMP. In accordance with Figure 13c,f, it can be observed that the mineralization efficiency for removing the TOC concentration was always lower than the removal efficiency for degrading SMP using DBHP, Dy_2_TmSbO_7_, BiHoO_3_, or N-T under visible light irradiation. The reason was attributed primarily to the generation and accumulation of the intermediate products for degrading SMP, indicating that the presence of the intermediate products often exhibited a negative correlation with the mineralization efficiency for removing the TOC concentration [92].

**Figure 14:**
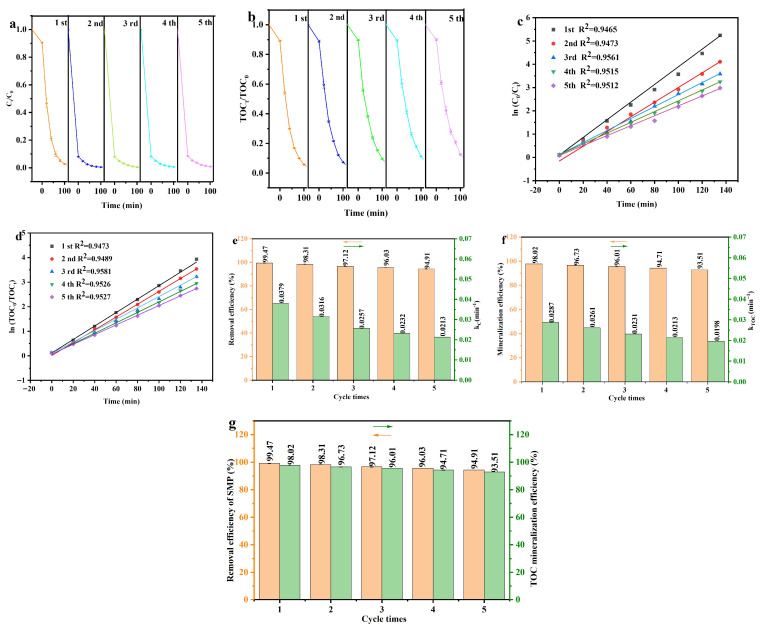
(**a**) Variety curves of SMP concentration relative to visible light irradiation time with DBHP for in the quintic cyclic PADA of SMP with DBHP; (**b**) evolution curves of TOC concentration relative to visible light irradiation time in quintic cyclic PADA of SMP with DBHP; (**c**) simulated profile of ln(*C*_0_/*C_t_*) as a function of visible light irradiation time in quintic cyclic PADA of SMP with DBHP; (**d**) simulated profile of ln(*TOC*_0_/*TOC_t_*) as a function of visible light irradiation time in quintic cyclic PADA of SMP with DBHP; (**e**) SMP degradation efficiency and the kinetic constants in quintic cyclic PADA of SMP under visible light irradiation with DBHP; (**f**) mineralization efficiencies for removing the TOC concn and the kinetic constants in quintic cyclic PADA of SMP under visible light irradiation with DBHP; (**g**) removal efficiencies of SMP and the mineralization efficiencies for removing the TOC concentration in quintic cyclic PADA of SMP under visible light irradiation with DBHP.

Figure 14a shows the variety curves of the SMP concentration relative to visible light irradiation time in quintic cyclic photocatalytic degradation of SMP with DBHP. Figure 14b depicts the evolution curves of the TOC concentration relative to the visible light irradiation time in the quintic cyclic PADA of SMP with DBHP. Figure 14c displays the simulated profile of ln(*C*_0_/*C_t_*) as a function of the visible light irradiation time in the quintic cyclic PADA of SMP with DBHP. Figure 14c revealed that the variation in ln(*C*_0_/*C_t_*) with the visible light irradiation time in the quintic cyclic PADA of SMP adhered to the first-order kinetic model. Figure 14d enunciates the simulated profile of ln(*TOC*_0_/*TOC_t_*) as a function of the visible light irradiation time in the quintic cyclic PADA of SMP with DBHP. Figure 14d revealed that the variation in ln(*TOC*_0_/*TOC_t_*) with the visible light irradiation time in the quintic cyclic PADA of SMP adhered to the first-order kinetic model. Figure 14e compares the SMP degradation efficiency and the kinetic constants which stem from the effect of the ln(*C*_0_/*C_t_*) on the visible light irradiation time in quintic cyclic PADA of SMP with DBHP. Figure 14f exhibits the mineralization efficiencies for removing the TOC concentration and the kinetic constants which derive from the effect of the ln(*TOC*_0_/*TOC_t_*) on the visible light irradiation time in the quintic cyclic PADA of the SMP with the DBHP. According to Figure 14a,e, the SMP degradation rate with DBHP after 135 min of visible light irradiation across the quintic consecutive cyclic PADA of SMP was 99.47%, 98.31%, 97.12%, 96.03%, or 94.90%. In light of Figure 14b,f, the removal rate of the TOC concentration with DBHP after 135 min of visible light irradiation across the quintic consecutive cyclic PADA of SMP was 98.01%, 96.73%, 96.01%, 94.72% or 93.51%. The above results indicate that DBHP has potential efficient recycle utilization capacity in the PADA of SMP under visible light irradiation. Figure 14g displays the removal efficiencies of SMP and the mineralization efficiencies for removing the TOC concentration in the quintic cyclic PADA of SMP under visible light irradiation with DBHP. As revealed in Figure 14g, DBHP has the ability to maintain a degradation efficiency of 94.90% and a mineralization efficiency of 93.51% after five consecutive cyclic PADA of SMP under visible light irradiation, indicating that DBHP has potential effectivity and stableness for removing SMP pollutant for wastewater remediation.

**Figure 15:**
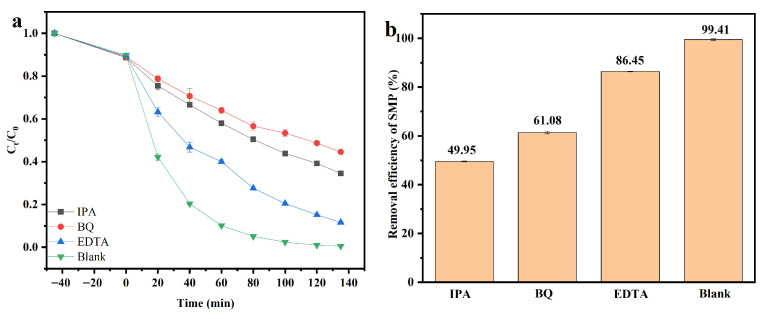
(**a**) Relationship between C_t_/C_0_ and visible light irradiation time in the PADA of SMP with DBHP, employing IPA, BQ, or EDTA as radical scavengers; (**b**) removal efficiency of SMP using IPA, BQ, or EDTA as radical scavengers.

Figure 15a illustrates the relationship between *C_t_*/*C*_0_ and visible light irradiation time in the photocatalytic degradation of SMP with DBHP, employing isopropanol (IPA), benzoquinone (BQ), or ethylenediaminetetraacetic acid (EDTA) as radical scavenger.

Figure 15b displays the removal efficiency of SMP in the PADA of SMP using IPA, BQ or EDTA as radicals scavenger under visible light irradiation with DBHP. According to Figure 15a,b, the IPA was utilized for capturing hydroxyl radicals, subsequently, the experimentative results showed that the removal rate of SMP using hydroxyl radicals was 34.09% after visible light irradiation of 135 min with the Dy_2_TmSbO_7_/BiHoO_3_ heterojunction as photocatalyst, meanwhile, BQ was used for capturing superoxide anions; as a result, the experimental conclusion indicated that the removal rate of SMP using superoxide anions was 44.31% after visible light irradiation of 135 min with the Dy_2_TmSbO_7_/BiHoO_3_ heterojunction as photocatalyst, additionally, the EDTA was utilized for capturing photogenerated holes; as a result, the experimental conclusion exhibited that the removal rate of SMP using photogenerated holes was 11.56% after visible light irradiation of 135 min with the Dy_2_TmSbO_7_/BiHoO_3_ heterojunction as photocatalyst. The above experimental results indicated that the oxidative capability of superoxide anions was higher than that of hydroxyl radicals or photogenerated holes, indicating that superoxide anions possessed the strongest removal efficiency for degrading SMP compared with hydroxyl radicals or photogenerated holes. Therefore, the oxidative capability of three radicals obeyed the descending order of superoxide anions > hydroxyl radicals > photogenerated holes.

**Figure 16:**
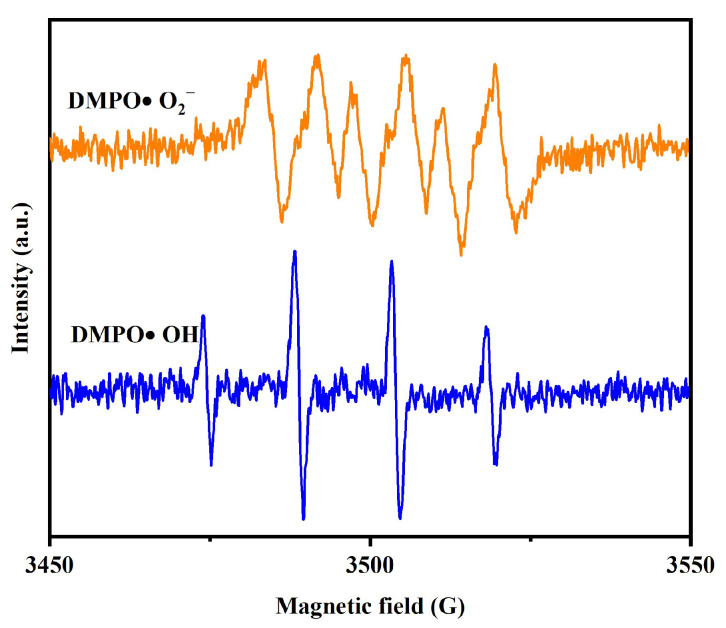
EPR spectrum for the DMPO•O_2_^−^ adduct or the DMPO•OH adduct in the PADA of SMP under visible light irradiation with DBHP.

Figure 16 presents the electron paramagnetic resonance (EPR) spectrum for the DMPO•O_2_^−^ adduct or the DMPO•OH adduct during the process of SMP under visible light irradiation with DBHP. As revealed in Figure 16, the signal peak of the hydroxyl radicals or the superoxide anions appeared in the PADA of SMP under visible light irradiation in the presence of DBHP; as a result, the EPR spectrum proved a clear DMPO•O_2_^−^ adduct signal peaks which were characterized by four well-defined peaks with an intensity ratio of 1:1:1:1, confirming that the superoxide anions was generated. Subsequently, the EPR spectrum which was displayed in Figure 16 indicated a four lines signal with an intensity ratio of 1:2:2:1, verifying that the DMPO•OH adduct was produced. The above findings demonstrated that both the hydroxyl radicals and the superoxide anions were simultaneously produced in the PADA of SMP under visible light irradiation with the DBHP. Additionally, the relative intensities of the EPR signals which stemmed from Figure 16 indicated that the generated amount of the hydroxyl radicals was more than that of the superoxide anions. The above results further confirmed and obeyed the results which originated from the radicals scavenger experiments, providing conclusive proof for the involvement of •O_2_^−^ and •OH in the PADA mechanism of SMP.

### 2.5. Comparison of the Photocatalytic Activity

Table 1 presents a removal efficiency comparison result of SMP using different catalysts under visible light irradiation. As revealed in Table 1, it was found that the degradation efficiency of SMP with the DBHP was 1.33 times that with TiO_2_ or 1.16 times that with β-Bi_2_O_3_/g-C_3_N_4_. The experimental results indicated that the DBHP exhibited the highest removal efficiency for degrading SMP compared with TiO_2_, β-Bi_2_O_3_/g-C_3_N_4_ or Dy_2_TmSbO_7_, demonstrating that the photocatalytic activity of the DBHP surpassed that of TiO_2_, β-Bi_2_O_3_/g-C_3_N_4_ or Dy_2_TmSbO_7_. The above analysis results confirmed the strong practical applicability of the DBHP which was synthesized in this study.

### 2.6. The Photocatalytic Mechanism of DBHP

**Figure 17:**
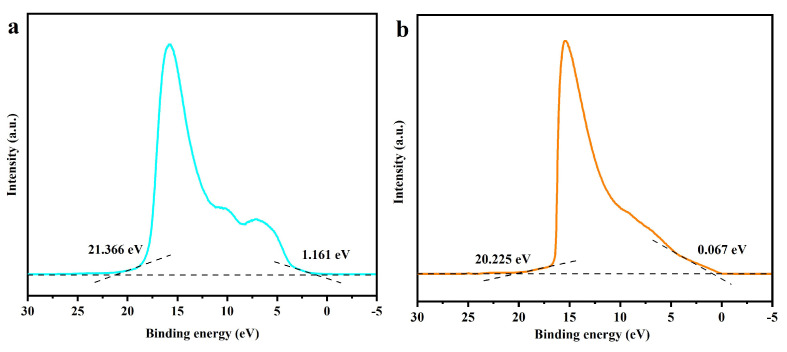
(**a**) The UPS spectrum of Dy_2_TmSbO_7_; (**b**) The UPS spectrum of BiHoO_3_.

Figure 17a represents the ultraviolet photoelectron spectroscopy (UPS) spectrum of Dy_2_TmSbO_7_. Figure 17b shows the UPS spectrum of BiHoO_3_. The measured onset value or the cutoff binding energy value of Dy_2_TmSbO_7_ was calculated to be 1.161 eV or 21.366 eV, respectively; simultaneously, the measured onset value or the cutoff binding energy value of BiHoO_3_ was calculated to be 0.067 eV or 20.225 eV. By considering the excitation energy of 21.2 eV, the ionization potential of Dy_2_TmSbO_7_ or BiHoO_3_ was accurately determined to be 2.926 eV or 1.042 eV. Therefore, the conduction band potential of Dy_2_TmSbO_7_ or BiHoO_3_ was computed to be 0.646 eV or −1.538 eV, respectively.

According to the literature, the photogenerated electrons located at the conduction band of CdS could directly transfer to the conduction band of AgI. The electrochemical potential of the conduction band for AgI was –0.47 eV, which was more negative than the standard potential of O_2_/•O_2_^−^ (–0.33 eV vs. NHE). This enabled the photogenerated electrons located at the conduction band of AgI to react with tje dissolved oxygen contained in water for generating •O_2_^−^. Meanwhile, the photogenerated holes which were produced on the valence band of AgI could transfer to the valence band of CdS. Since the ionization potential of the valence band for CdS was +1.65 eV, which was more negative than the standard potential of •OH/OH^−^ (+2.38 eV vs. NHE); as a result, the photogenerated holes located at the valence band of CdS could not oxidize OH^−^ to form •OH [95]. The type-II mechanism which was observed in our study is consistent with the type-II mechanism that found in the literature [95].

**Figure 18:**
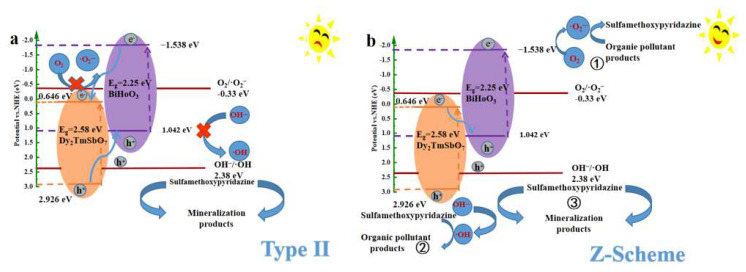
The possible photodegradation mechanism of SMP during the PADA of SMP under visible light irradiation with DBHP: (**a**) conventional type II; (**b**) direct Z-scheme.

As revealed in Figure 18a, in the traditional type-II migrating mode, the photogenerated electrons located at the conduction band of BiHoO_3_ were entirely transferred to the conduction band of Dy_2_TmSbO_7_. The electrochemical potential of the conduction band for Dy_2_TmSbO_7_ was 0.646 eV, which was more positive than the standard potential of O_2_/•O_2_^−^ (−0.33 eV vs. NHE). Consequently, the above descriptive inference could not drive the photogenerated electrons to interact with dissolved oxygen which was contained in water for forming •O_2_^−^. Simultaneously, the photogenerated holes (h^+^), which were located at the valence band of Dy_2_TmSbO_7_, completely migrated to the valence band of BiHoO_3_. However, the ionization potential of the valence band for BiHoO_3_ was 1.042 eV, which was more negative than the standard potential of OH^−^/•OH (2.38 eV vs. NHE). Therefore, the photogenerated holes located at the valence band of BiHoO_3_ could not oxidize OH^−^ to generate •OH, which possess high oxidative ability. Under this type-II transfer mechanism, the high reactive superoxide radicals or hydroxyl radicals could not be produced in this study. These findings contradict the experimental detection results of •O_2_^−^ and •OH, which were secured from the trapping experiments and the EPR analysis results.

Therefore, another direct Z-scheme heterojunction model is proposed in Figure 18b. In accordance with the direct Z-scheme heterojunction model which was displayed in Figure 18b, it was expected that the photogenerated electrons would migrate from the conduction band position of Dy_2_TmSbO_7_ which owned potential of 0.646 eV to the valence band position of the BiHoO_3_ which possessed potential of 1.042 eV, thereby the immediate recombination of the photogenerated electrons and the photogenerated holes which derived from the low oxidative potential and low reductive potential within the Dy_2_TmSbO_7_/BiHoO_3_ heterostructure was promoted. In order to enhance the removing rate for degrading SMP, the PADA mechanism of SMP should maintain the high oxidative potential and the high reductive potential which originated from DBHP. As revealed in the PADA mechanism ① of Figure 18b, the conduction band potential of the BiHoO_3_ was −1.538 eV which was more negative than −0.33 eV which represented the standard potential of O_2_/•O_2_^−^. The above high reductive potential was sufficient to induce interaction which derived from the photogenerated electrons and oxygen for forming •O_2_^−^. As illustrated in the PADA mechanism ② of Figure 18b, the valence band potential of Dy_2_TmSbO_7_ was 2.926 eV which was more positive than 2.38 eV that represented the standard potential of OH^−^/•OH; consequently, the photogenerated holes which occurred in the valence band position of Dy_2_TmSbO_7_ could oxidize OH^−^ for generating highly reactive •OH. As depicted in the PADA mechanism ③ of Figure 18b, the photogenerated holes which arose in the valence band position of Dy_2_TmSbO_7_ or the BiHoO_3_ possessed inherent desirable oxidation potential and might act as catalytic reaction radicals for the oxidation and subsequent degradation of SMP. Thus, the generated •O_2_^−^, •OH or h^+^ collectively provided contribution for the elimination of SMP. In conclusion, the above analytic results were consistent with the experimental results which originated from the trapping scavenger experiments and the EPR tests. Based on above analysis results, the direct Z-scheme heterojunction structure successfully explains the photocatalytic mechanism that is observed within the DBHP; meanwhile, the DBHP exhibited great potential for removing pollutants from wastewater.

**Figure 19:**
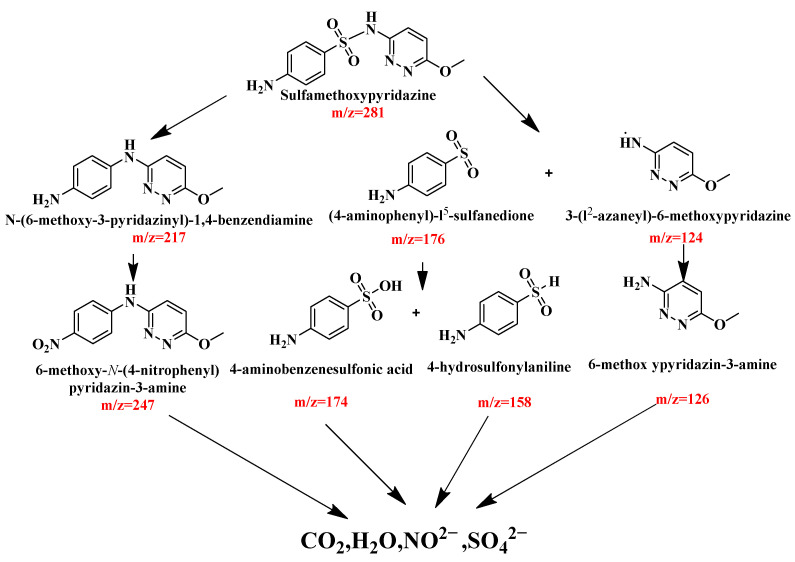
The viable photodegradation pathway for SMP in the PADA of SMP under visible light irradiation with DBHP.

In accordance with the extant literature and the results deriving from the liquid chromatography-mass spectrometry (LC-MS), Figure 19 shows the viable photodegradation pathway for SMP during the PADA of SMP (*m*/*z* = 277) under visible light irradiation with the DBHP. It could be found from Figure 19 that two distinct PADA mechanisms had been identified according to the detected mesial products for degrading SMP in the PADA process under visible light irradiation with the DBHP. The first pathway, a key route for the sulfonamide antibiotic oxidation, arose from SO_2_ elimination; as a result, the *N*-(6-methoxy-3-pyridinyl)-1,4-phenylenediamine (*m*/*z* = 217) formed. Subsequent oxidation of the -NH_2_ group produced 6-methoxy-*N*-(4-nitrophenyl) pyridin-3-amine (*m*/*z* = 247). The second pathway involved the oxidative disunion of the sulfonamide bond (S-N) of SMP using reactive species, subsequently, two benzenesulfonic acid residues which were identified as (4-aminophenyl)-l^5^-sulfanedione (*m*/*z* = 176) and 3-(l^2^-azaneyl)-6-methoxypyridazine (*m*/*z* = 124) were produced. Concurrently, these resultant sulfonic acid residue subsequently underwent hydrolysis and (or) further oxidation; as a result, the formation of the 6-methoxypyridin-3-amine (*m*/*z* = 126), 4-aminobenzenesulfonic acid (*m*/*z* = 174) and 4-(methylsulfonyl)aniline (*m*/*z* = 158) was realized. Meanwhile, the loss and subsequent oxidation of the sulfanilamide would lead to the formation of 6-methylpyridin-3-amine. Ultimately, 6-methoxy-*N*-(4-nitrophenyl) pyridazin-3-amine (*m*/*z* = 247), 4-aminobenzenesulfonic acid (*m*/*z* = 174), 4-methylsulfonylaniline (*m*/*z* = 158) and 6-(methoxyphenyl)pyridin-3-amine (*m*/*z* = 126) underwent further degradation; as a result, the above four intermediate products would be turned into carbon dioxide (CO_2_), water (H_2_O), and inorganic anions, which included sulfate (SO_4_^2−^) and nitrite (NO_2_^−^) [96,97].

## 3. Experimental Section

### 3.1. Materials and Reagents

Dy_2_O_3_ (purity 99.99%), Tm_2_O_3_ (purity 99.99%), Sb_2_O_5_ (purity 99.99%), Bi_2_O_3_ (purity 99.99%), Ho_2_O_3_ (purity 99.99%), ethylenediaminetetraacetic acid (EDTA, C_10_H_16_N_2_O_8_, purity = 99.5%), isopropanol (IPA, C_3_H_8_O, purity ≥ 99.5%), benzoquinone (BQ, C_6_H_4_O_2_, purity ≥ 98.0%), tetrabutyl titanate (C_16_H_36_O_4_Ti, purity ≥ 99.9%), octanol (C_8_H_18_O, purity ≥ 98.0%), absolute ethanol (C_2_H_5_OH, purity ≥ 99.5%), sulphamethoxypyridazine (C_11_H_12_N_4_O_5_S, purity ≥ 99.0%), and ultrapure water (18.25 MU-cm). The above chemical solutions were purchased from Aladdin Group Chemical Reagent Co., Ltd. (Shanghai, China).

### 3.2. Preparation Method of Dy_2_TmSbO_7_

Dy_2_TmSbO_7_ was prepared using the high-temperature solid-phase calcination method. Dy_2_O_3_, Tm_2_O_3_, and Sb_2_O_5_ were used as raw materials. The above raw materials were mixed according to the stoichiometric mole ratio of n_1_(Dy_2_O_3_):n_2_(Tm_2_O_3_):n_3_(Sb_2_O_5_) = 2:1:1, fully mixed, then ground, and dried in a ball mill, pressed into tablets in an alumina crucible, and placed in a high-temperature sintering furnace for sintering. The temperature was raised from room temperature to 750 °C, then kept at 750 °C for 240 min, raised from 250 °C to 1180 °C, then kept at 1180 °C for 1800 min, and finally cooled from 1180 °C to room temperature. The tablet sample was put into an agate mortar to be crushed and ground for 90 min, and, finally, the Dy_2_TmSbO_7_ powder was obtained.

### 3.3. Preparation Method of BiHoO_3_

A novel BiHoO_3_ photocatalyst was synthesized via a high-temperature solid-state sintering method. Bi_2_O_3_ and Ho_2_O_3_ were used as raw compounds with a purity of 99.99%. The Bi_2_O_3_ oxide and the Ho_2_O_3_ oxide were first thoroughly mixed with a stoichiometric mole ratio of n_4_(Bi_2_O_3_):n_5_(Ho_2_O_3_) = 1:1, then ball milled, dried, pressed into pellets within an alumina crucible, and transferred to a high-temperature sintering furnace for the heat-agglomerating process. The detailed heat-agglomerating technology was as follows: the mixed powder was heated from room temperature to 400 °C and held at 400 °C for 240 min, then heated from 400 °C to 720 °C, held at 720 °C for 600 min, heated from 720 °C to 1150 °C, held at 1150 °C for 1560 min, and, ultimately, the mixed powder was cooled down from 1150 °C to room temperature. The sintered pellets were subsequently crushed and ground in an agate mortar for 90 min to obtain the final BiHoO_3_ powder.

### 3.4. Manufacturing Method for the N-Doped TiO_2_

The complete manufacturing method of the nitrogen-doped TiO_2_ is provided in the Supplementary Materials (Section S1).

### 3.5. Preparation of the Dy_2_TmSbO_7_/BiHoO_3_ Heterojunction Photocatalyst

DBHP was synthesized by the ultrasound-assisted solvothermal method. A total of 0.012 mol of Dy_2_TmSbO_7_ and 0.012 mol BiHoO_3_ were mixed in octanol and underwent ultrasonic treatment in an ultrasound bath for 300 min. Subsequently, the mixture was magnetically stirred. The mixture was maintained at 190 °C to promote the deposition of the Dy_2_TmSbO_7_ nanoparticles onto the surface of the BiHoO_3_ nanoparticles. After reaching room temperature, the resulting product was separated by centrifugation, followed by multiple ethanol washes for ensuring thorough purification. The purified powder was dried in a vacuum oven at 110 °C and then the above purified powder was placed for 90 min to obtain the DBHP.

### 3.6. Photoelectrochemical Measurements

The electrochemical impedance spectroscopy (EIS) measurements were executed using a CHI_6_60D electrochemical workstation (Chenhua Instruments Co., Ltd., Shanghai, China) with a three-electrode system configuration. A platinum sheet served as the counter electrode; concurrently, a silver/silver chloride (Ag/AgCl) electrode served as the reference electrode; moreover, the prepared glassy carbon electrode served as the working electrode. The electrolyte employed was an aqueous sodium sulfate solution which had a concentration of 0.3 mol/L. Light simulation during the experiments was achieved using a 500 W xenon lamp, which was equipped with a 420 nm cut-off filter.

The preparation process of the working electrode was as follows: 8 mg of the synthesized material was dispersed in a mixture which involved 640 µL ethanol, 960 µL ultrapure water, and 32 µL perfluorosulfonic acid solution; subsequently, the above mixture underwent an ultrasonication treatment for 40 min. Ultimately, 8 µL of the abovementioned suspension was uniformly added drop by drop onto the surface of the polished glassy carbon electrode; subsequently, the abovementioned suspension was dried under infrared light irradiation.

### 3.7. Feature Description

The feature description was put in the Supplementary Materials (Section S2).

### 3.8. Explanation of the Experimental Setup and Procedures

The explanation of the experimental setup and procedures was put in the Supplementary Materials (Section S3).

## 4. Conclusions

On the whole, a direct Z-scheme heterojunction photocatalyst Dy_2_TmSbO_7_/BiHoO_3_ is resoundingly synthesized by employing a straightforward ultrasonic-assisted solvothermal method. The findings revealed a distinct Z-scheme charge transfer mechanism that existed between the Dy_2_TmSbO_7_ and the BiHoO_3_; as a result, the above distinct Z-scheme charge transfer mechanism could significantly enhance the separation efficiency of the photogenerated electrons and the photogenerated holes. In addition, the formation of this Z-scheme heterojunction substantially improved the redox capacity of DBHP. The photoelectrochemical tests demonstrated that the optimized DBHP exhibited remarkable separating efficiency in connection with the photogenerated electrons and the photogenerated holes. When SMP was employed as a model pollutant under visible light irradiation, the DBHP displayed exceptional photodegradation activity, compared with Dy_2_TmSbO_7_, BiHoO_3_, or N-T. The removal rate of SMP after visible light irradiation of 135 min with DBHP was 1.15 times that using the Dy_2_TmSbO_7_, 1.29 times that using the BiHoO_3_, or 2.60 times that using the N-T. The EPR analysis results elucidated that the oxidative capability of three radicals obeyed the descending order of •O_2_^−^ > •OH > h^+^ in the PADA of SMP under visible light irradiation. Additionally, the possible degradation pathways and the degradation mechanisms for SMP were proposed. In conclusion, DBHP possesses significant application potential for removing the antibiotic contaminants contained in pharmaceutical wastewater.

## Figures and Tables

**Table 1:** The removal efficiency comparison result of the SMP by using different catalysts during the photodegradation process of the SMP under visible light irradiation.

Photocatalyst	Incident Light	Irradiation Time (min)	Name of Antibiotic	Removal Rate (%)	Reference
TiO_2_	The light of LED 398 nm		Sulphamethoxypyridazine	75	[93]
β-Bi_2_O_3_/g-C_3_N_4_	Visible light	180	Sulphamethoxypyridazine	85.5	[94]
Dy_2_TmSbO_7_	Visible light	135	Sulphamethoxypyridazine	86.31	This study
DBHP	Visible light	135	Sulfathiazole	99.47	This study

## Data Availability

Data are contained within the article.

## Supplementary Materials

The following supporting information can be downloaded at: https://www.mdpi.com/article/10.3390/molecules31010024/s1. Figure S1: The N_2_ adsorption isotherms of DBHP, Dy_2_TmSbO_7_, and BiHoO_3_; Figure S2: The three-dimensional fluorescence spectra of SMP or the intermediate degradation products during the photocatalytic degradation process of SMP with DBHP under visible light irradiation; Table S1: The architecture dimension of Dy_2_TmSbO_7_ fabricated by the solid-state calcination method; Table S2: The architecture dimension of BiHoO_3_ fabricated by the solid-state calcination method; Table S3: The various chemical bonds and their corresponding peaks deriving from the FTIR spectra of the DBHP, Dy_2_TmSbO_7_, and BiHoO_3_; Table S4: The various chemical bonds and their corresponding peaks deriving from the Raman spectra of the DBHP, Dy_2_TmSbO_7_, and BiHoO_3_; Section S1: Preparation of Nitrogen-Doped TiO_2_; Section S2: Feature description; Section S3: Explanation of the experimental setup and procedures.
