# Supplementary material for: Design and Synthesis of Dy2TmSbO7/BiHoO3 Heterojunction: The Mechanism and Application for Photocatalytic Degradation of Sulphamethoxypyridazine
Source: Molecules. 2025 Dec 22;31(1):24. doi: 10.3390/molecules31010024 (PMC12787114; doi:10.3390/molecules31010024)
Supplement: Supplementary file 1 [file molecules-31-00024-s001.zip › molecules-4030094-supplementary.pdf]

Design and Synthesis of $Dy_2TmSbO_7/BiHoO_3$ Heterojunction: The Mechanism and Application for photocatalytic degradation of Sulphamethoxypyridazine

Jingfei Luan[1,2*], Minghe Ma[1], Liang Hao[1], Hengchang Zeng[1], Anan Liu[1]

1 School of Physics, Changchun Normal University, Changchun 130032, China; 13251704137@139.com (M.M.); 19845486007@139.com(L.H.); zenghc23@mails.jlu.edu.cn (H.Z.); ananliu2001@outlookcom (A.L.).
2 State Key Laboratory of Pollution Control and Resource Reuse, School of the Environment, Nanjing University, Nanjing 210093, China
* Correspondence: jfluan@nju.edu.cn; Tel.: +86-199-5193-9498

Table S1. The architecture dimension of the Dy_2TmSbO_7 photocatalyst fabricated by the solid-state calcination method.

Atom	x	y	z	Occupation factor
Dy	0	0	0	1
Tm	0.5	0.5	0.5	0.5
Sb	0.5	0.5	0.5	0.5
O(1)	-0.175	0.125	0.125	1
O(2)	0.125	0.125	0.125	1

Table S2. The architecture dimension of the $BiHoO_3$ photocatalyst fabricated by the solid-state calcination method.

Atom	x	y	z	Occupation factor
Bi	0	0	0	0.5
Ho	0	0	0	0.5
O	0.25	0.25	0.25	0.75

Table S3. The various chemical bonds and their corresponding peaks deriving from the FTIR spectra of the DBHP, Dy_2TmSbO_7 and $BiHoO_3$.

Chemical bonds	Peak positions of chemical bonds	References
Bi-O	413 cm^{-1}	[57]
Ho-O	598 cm^{-1}	[58]
Dy-O	434 cm^{-1}	[59]
Tm-O	660 cm^{-1}	[60]
Sb-O-Sb	608 $cm^{-1}$and 763 cm^{-1}	[61]
H-O-H	1584 cm^{-1}	[63]
O-H	1389 cm^{-1}	[64]

Table S4. The various chemical bonds and their corresponding peaks deriving from the Raman spectra of the DBHP, Dy_2TmSbO_7 and $BiHoO_3$.

Chemical bonds	Peak positions of chemical bonds	References
Dy-O	733 cm^{-1}	[65]
Tm-O	392 cm^{-1}	[66]
Bi-O	627 cm^{-1}	[67]
Ho-O	292 cm^{-1}	[68]

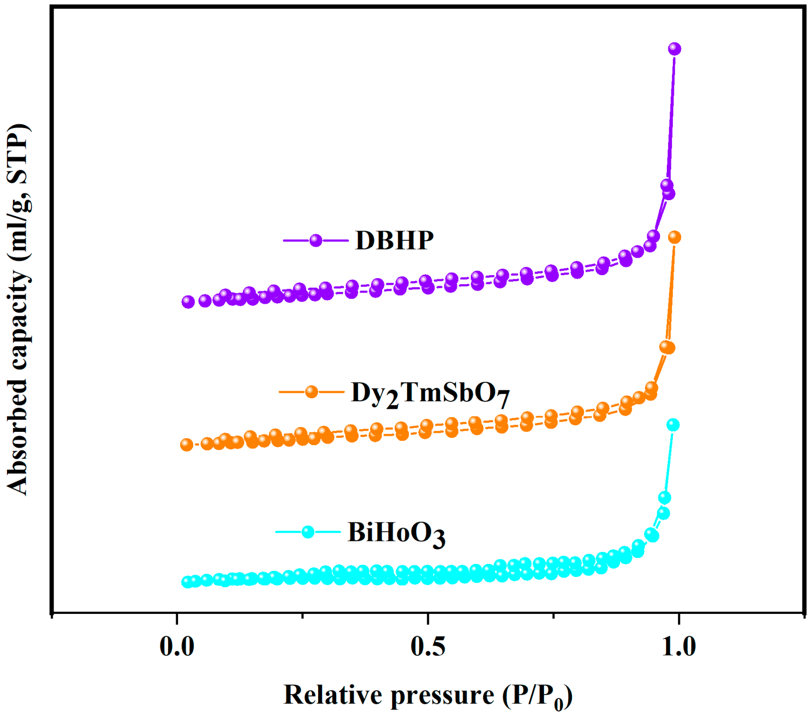

Figure S1. The N_2 adsorption isotherms of DBHP, Dy_2TmSbO_7 and $BiHoO_3$.

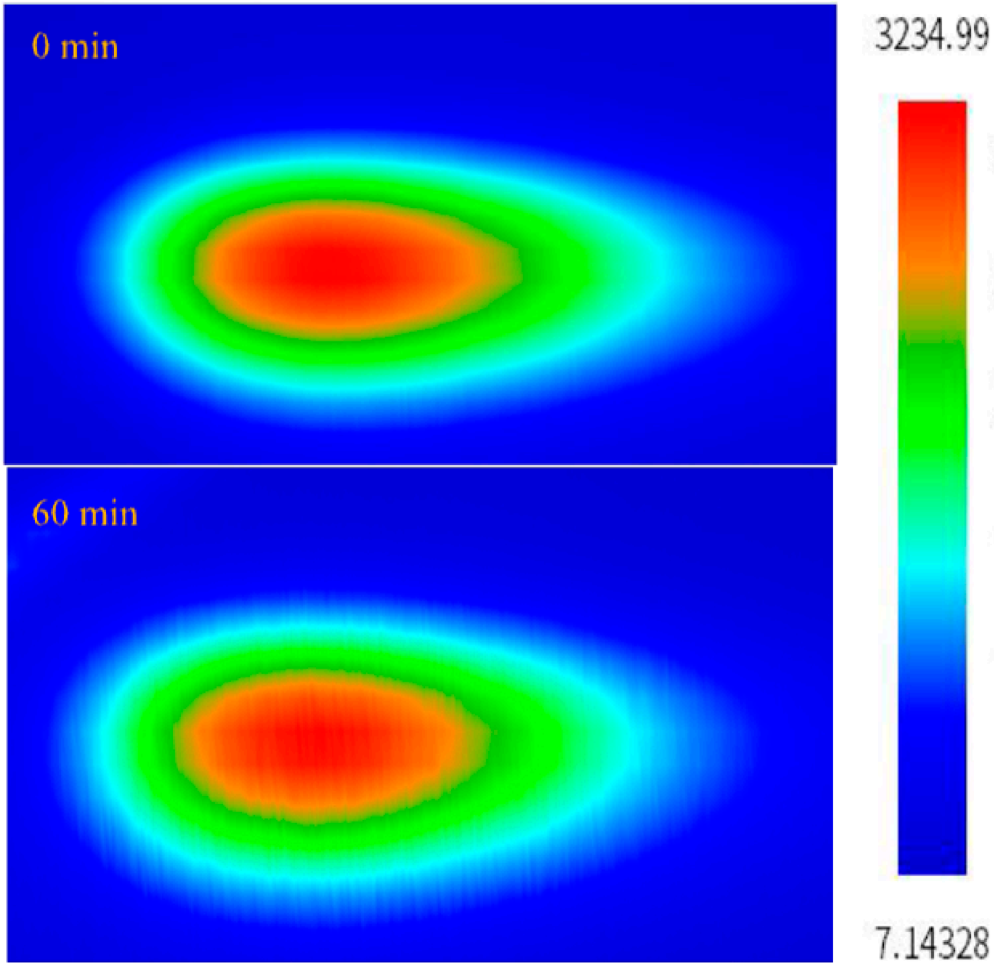

Figure S2. The three-dimensional fluorescence spectra of the SMP or the intermediate degradation products during the photocatalytic degradation process of the SMP with DBHP under VLIDN.

Section S1: Manufacturing method of N-doped TiO_2

A specified amount of tetrabutyl titanate was mixed with absolute alcohol for obtaining solution A. Under the condition of magnetic stirring, the solution A was dropwise added to the solution B which was composed of glacial acetic acid, double-distilled water and absolute alcohol. Subsequently, the mixed solution was stirred vigorously for 30 minutes, then different amounts of 1mol/L ammonia solution were added gradually to the mixture with a N/Ti molar ratio of 8%. Afterwards, the stirring process was continued for another 120 minutes, and the resulting mixture was should keep equilibration for 1 day at room temperature. Subsequently, above resulting mixture was transferred to a drying oven and was heated at 105°C. Next step, the solid mixture was taken out from the drying oven and was ground, then the the solid mixture was calcined at 400°C to obtain the solid powder. Ultimately, above solid powder was ground again in a high-temperature furnace for 120 minutes, as a result, the nitrogen-doped titanium dioxide powder was obtained.

Section S2: Feature Description

In this paper, various characterization instruments such as X-ray diffractometer, TEM, X-ray photoelectron spectrum, Fourier transform infrared spectrometer, Raman spectrometer and ultraviolet visible diffuse reflectance spectrophotometer (UV-Vis DRS) were used for investigating the anatomical characteristics of pure phase Dy_2TmSbO_7 and pure phase $BiHoO_3$ which were prepared by using the solid-phase calcination method. In addition, the removal rate of the SMP by using pure phase Dy_2TmSbO_7, pure phase $BiHoO_3$, the N-T or the DBHP during the photocatalytic degradation process of the SMP under the condition of VLIDN was also determined. The crystallographic structure data could be obtained by using the X-ray powder diffractometer (XRD-6000 diffractometer, Shimadzu, Kyoto, Japan). The TEM (Talos F200X G2 instrument, Thermo Fisher Scientific, Waltham, Massachusetts, USA) was utilized for analyzing the surface morphology, high-resolution lattice fringe image and element mapping image of the DBHP. The X-ray photoelectron spectrometer (XPS, PHI 5000 Versa Probe instrument, ULVAC-PHI, Chigasaki, Japan) was utilized for analyzing the surface elemental composition and chemical valent state of the photocatalysts. The Fourier transform infrared (FTIR) spectrum analysis results were conducted by using a Fourier transform infrared spectrometer (WQF-530A, Beifang Rayleigh, Beijing, China) for revealing the information such as the molecular structure, chemical bond type or functional group composition of the photocatalysts. The Raman spectrum analysis results were performed by using the Raman spectrometer (inVia Reflex, Renisshawplx, London, UK) for investigating the chemical structure, the lattice dynamics and the molecular interactions of the photocatalysts. The optical properties of the photocatalyst samples were evaluated by using an ultraviolet visible diffuse reflectance spectrometer (UV3600, DRS, Kyoto, Japan). In addition, the EDS analysis results were conducted for determining the elemental composition distribution and the composition content of the DBHP. The photoluminescence (PL) spectrum and the fluorescence lifetime of the photocatalysts were measured by using a fluorescence spectrometer (FLS1000, Edinburgh Instruments, Edinburgh, UK). The Thermo Fisher Scientific Escalab 250 xi instrument (Waltham, Massachusetts, USA) was used as ultraviolet photoelectron spectroscopy (UPS), as a result, the ionization potential of the valence band for the Dy_2TmSbO_7 or the $BiHoO_3$ was obtained. Finally, the electron paramagnetic resonance (EPR) spectrometer which was equipped with a Bruker instrument (A300, Karlsruhe, Germany) was employed for detecting the active radicals during the photocatalytic process of the SMP with the DBHP under VLIDN.

Section S3: Explanation of the Experimental Setup and Procedures

Under VLIDN, the photocatalytic degradation (photocatalytic degradation) experiments for the SMP were conducted by using a photocatalytic reactor (CELLB70, China Education Jin Guang Technology Co., Ltd., Beijing, China). The required light source was generated by a 500

W xenon lamp which was equipped with a 420 nm cut-off filter. 12 quartz tubes were utilized as to each experiment, simultaneously, each quartz tube contained the reaction solution of 40 mL, as a result, a total reaction system volume of 480 mL was gained. The Dy_2TmSbO_7, the $BiHoO_3$, the N-T or DBHP was applied at a dosage of 0.8 g/L for the photocatalytic degradation of the SMP. The initial concentration of the target pollutant SMP which was contained in the wastewater solution was measured to be 0.032 mmol/L.

During the photocatalytic degradation process of the SMP, 5 mL solution sample was periodically collected for analyzing subsequent concentration variation of the SMP or subsequent TOC concentration variation with an interval of 20 minutes. The reaction suspension which contained the photocatalyst powder was first filtered through 0.22 μm polyethersulfone membranes for removing the photocatalyst samples, subsequently, every 5 mL sample underwent centrifugation with 6700 rpm for separating the clear liquid which as utilized for next analysis, concurrently, the remaining SMP concentration was determined by using a high-performance liquid chromatograph (HPLC, Agilent 200, Agilent Technologies Inc., Palo Alto, California, USA). During the photodegradation process of the SMP, 10 μL clear supernatant which derived from above centrifugation was injected into the HPLC at a flow rate of 1 mL/min for the concentration analysis of the SMP or the intermediate product. Prior to the VLIDN, the photocatalytic degradation system which contained the nano photocatalyst and the SMP was stirred under the dark condition for 45 minutes, as a result, the photocatalytic degradation system reached adsorption/desorption equilibrium.

In order to evaluate the mineralization efficiency of the TOC concentration during the photocatalytic degradation process of the SMP, a TOC analyzer (Sievers 500 RL, Veolia, Brea, California, USA) was utilized for determining the variation of the TOC concentration during the photodegradation process of the SMP. Potassium hydrogen phthalate ($KHC_8H_4O_4$) or anhydrous sodium carbonate was served as reference reagent. By using the potassium hydrogen phthalate, the calibration standards were established, as a result, the carbon concentration range was covered from 0 mg/L to 100 mg/L. 12 samples were used for the TOC assessment, meanwhile, each sample was composed of the reaction solution of 40 mL.

For the sake of the catalyst recovery during the cyclic photodegradation experiment, the reaction solution was transferred to the centrifuge tubes, concurrently, the subsequent centrifugation would be realized after the completion of the photocatalytic degradation for the SMP. Above centrifuge tubes underwent high-speed centrifugation with 1200 rpm, subsequently, the solid photocatalyst was effectively precipitated at the bottom of the tubes. After catalyst precipitation, the supernatant was carefully decanted for avoiding disturbing the settled photocatalyst. The resulting precipitate was then subjected to further centrifugation with pure water or ethanol for removing any residual reactants or products which adhered to the photocatalyst. The washed photocatalyst underwent an additional round of centrifugation for ensuring thorough cleaning, as a result, the washing solvent was removed. The collected photocatalyst was transferred to a drying dish or appropriate container for eliminating the residual moisture or solvent. Finally, the dried photocatalyst was ready for property characterization or was reused in the subsequent photocatalytic experiments.

In order to accomplish the calibration experiments of the targeting intermediate reactants, a liquid chromatograph-mass spectrometer (LC-MS, Thermo Quest LCQ Duo, Thermo Fisher Scientific, Waltham, Massachusetts, USA) was employed. During the photocatalytic degradation process of the SMP, a beta basic-C18 HPLC column (150 mm × 2.1 mm, 5 μm pore size, Thermo Fisher Scientific, Waltham, Massachusetts, USA) was utilized. During the photocatalytic degradation process of the SMP, the resulting reaction solution of 20 μL was automatically injected into the LC-MS system. The mobile phase which was used in the LC-MS system consisted of a mixture which was composed of 60% methanol and 40% ultrapure water. The scanning range of the mass spectrometer was set from 50 m/z to 400 m/z.

The incident photon flux after VLIDN was measured to be 4.76×10^{-6} Einstein·L^{-1}·s^{-1} by using a radiometer. The photon flux could be changed by adjusting the distance which was between the bioreactor and the light source. The PHEY was calculated by using the following formula:

$$\phi = R/I_0$$

the ϕ represented the PHEY (%), meanwhile, the R was the degradation rate of the SMP, moreover, the I_0 was the incident photon flux.
